# Cryo-EM structure of TFIIH/Rad4–Rad23–Rad33 in damaged DNA opening in nucleotide excision repair

**DOI:** 10.1038/s41467-021-23684-x

**Published:** 2021-06-07

**Authors:** Trevor van Eeuwen, Yoonjung Shim, Hee Jong Kim, Tingting Zhao, Shrabani Basu, Benjamin A. Garcia, Craig D. Kaplan, Jung-Hyun Min, Kenji Murakami

**Affiliations:** 1 Department of Biochemistry and Biophysics, Perelman School of Medicine, University of Pennsylvania, Philadelphia, PA USA; 2 Biochemistry and Molecular Biophysics Graduate Group, Perelman School of Medicine, University of Pennsylvania, Philadelphia, PA USA; 3 Penn Center for Genome Integrity, Perelman School of Medicine, University of Pennsylvania, Philadelphia, PA USA; 4 Department of Chemistry and Biochemistry, Baylor University, Waco, TX USA; 5 Epigenetics Institute, Department of Biochemistry and Biophysics, Perelman School of Medicine, University of Pennsylvania, Philadelphia, PA USA; 6 Department of Biological Sciences, University of Pittsburgh, Pittsburgh, PA USA

**Keywords:** Structural biology, Nucleotide excision repair, Cryoelectron microscopy

## Abstract

The versatile nucleotide excision repair (NER) pathway initiates as the XPC–RAD23B–CETN2 complex first recognizes DNA lesions from the genomic DNA and recruits the general transcription factor complex, TFIIH, for subsequent lesion verification. Here, we present a cryo-EM structure of an NER initiation complex containing Rad4–Rad23-Rad33 (yeast homologue of XPC–RAD23B–CETN2) and 7-subunit coreTFIIH assembled on a carcinogen-DNA adduct lesion at 3.9–9.2 Å resolution. A ~30-bp DNA duplex could be mapped as it straddles between Rad4 and the Ssl2 (XPB) subunit of TFIIH on the 3' and 5' side of the lesion, respectively. The simultaneous binding with Rad4 and TFIIH was permitted by an unwinding of DNA at the lesion. Translocation coupled with torque generation by Ssl2 and Rad4 would extend the DNA unwinding at the lesion and deliver the damaged strand to Rad3 (XPD) in an open form suitable for subsequent lesion scanning and verification.

## Abstract (web summary)

The conserved eukaryotic nucleotide excision repair (NER) pathway protects the genome from a wide variety of environmentally induced DNA lesions. Here, the authors provide insights into how NER is initiated on lesions by determining the cryo-EM structure of the yeast TFIIH/Rad4–Rad23-Rad33 complex bound to a DNA containing a single carcinogen-DNA adduct.

## Introduction

Genomic DNA is continuously being damaged by various endogenous and exogenous genotoxic agents. If left unrepaired, DNA lesions can impair cellular functions and lead to mutations that cause various disorders such as cancers (reviewed in refs. ^1–3^). Among various DNA damage repair pathways, nucleotide excision repair (NER) is highly conserved among eukaryotes and serves to repair a wide variety of environmentally induced lesions, including intra-strand cross-links induced by the sunlight (ultraviolet irradiation) or chemical crosslinkers (e.g., cisplatin), and various bulky adducts induced by environmental carcinogens present in fossil fuel combustion, cigarette smoke, cooked meat, etc.^4^.

Impaired NER can decrease cell survival after UV exposure in yeast and other cell lines^5^. In humans, mutations in key NER proteins underlie genetic disorders such as xeroderma pigmentosum (XP), Cockayne syndrome, and trichothiodystrophy^6^. Patients with XP are hypersensitive to UV light and exhibit >1000-fold increase in the risk of skin cancers including melanomas and squamous cell carcinomas. Cockayne syndromes results in premature aging with neurological abnormalities.

NER lesions can be recognized via two distinct sub-pathways, either in global genome NER (GG-NER) or in transcription-coupled NER (TC-NER) (reviewed in refs. ^7,8^). In TC-NER, lesions are recognized when RNA polymerase II stalls at a lesion during transcription elongation, while in GG-NER, the XPC protein in complex with RAD23B and centrin 2 (CETN2) functions as the main sensor that recognizes diverse NER lesions. Lesion-bound XPC recruits TFIIH, a conserved general transcription factor required for both transcription initiation and NER^9–13^, which then scans the damaged strand and verifies the presence of a bulky lesion, aided by XPA and RPA^14,15^. Subsequently, the lesion-containing single-stranded DNA (24−32 nucleotides) is excised by XPF-ERCC1 and XPG endonucleases on the 5' and 3'-sides of the lesion, respectively; the resulting gap is resynthesized by DNA polymerases with nick sealing by DNA ligases.

The key to the remarkable versatility of GG-NER lies in its unique 2-step initiation process involving XPC and TFIIH, which bypasses the need to rely on any specific structure of a lesion^16,17^. As a first step, XPC locates a damaged site by sensing local DNA destabilization/distortion induced by a lesion, rather than the lesion structure itself^18–21^. Crystallographic studies of Rad4–Rad23 and yeast homologues of XPC–RAD23B provide structural insights into such an indirect recognition mechanism^22–24^. Rad4 interacts with DNA through its four consecutive domains: the transglutaminase domain (TGD) and the β-hairpin domain 1 (BHD1) bind to a ~11-bp undamaged segment of the DNA duplex, whereas BHD2 and BHD3 together bind to a 4-bp segment harboring the damage. This binding results in an ‘open’ DNA conformation where the damaged site is locally unwound and two damage-containing nucleotide pairs are flipped out of the DNA duplex, while a β-hairpin from BHD3 plugs into the gap in the duplex to stabilize such conformation^25,26^. Notably, BHD2 and BHD3 selectively bind to the flipped-out nucleotides from the undamaged strand and do not make direct contacts with the damaged nucleotides, thereby enabling indirect readout of various lesions regardless of the specific structures. Further kinetic and single-molecule studies have revealed that Rad4 performs fast diffusional search while probing the DNA structural integrity that involves untwisting^24–26^.

As a second step of the NER initiation process, the lesion-bound XPC recruits TFIIH and TFIIH verify the presence of a genuine NER lesion (a bulky damage) through the function of the XPD helicase^27,28^. For instance, a 2- to 3-bp mismatched site can be specifically bound to XPC but is not repaired by NER, due to failure in this verification step^16,17^. The 10-subunit holoTFIIH consists of a 7-subunit core complex (hereafter coreTFIIH) and a 3-subunit Cdk-activating kinase module (CAK; TFIIK in yeast)^29,30^. The coreTFIIH contains two helicase-family ATPase subunits: XPB(Ssl2) and XPD(Rad3) in human(yeast), and 5 nonenzymatic subunits: p62(Tfb1), p52(Tfb2), p44(Ssl1), p34(Tfb4), and p8(Tfb5). One ATPase, XPB(Ssl2), shows preference for double-stranded DNA (dsDNA) over single-stranded DNA (ssDNA) and is active as an ATP-dependent DNA translocase in both transcription and NER; the other ATPase subunit, XPD(Rad3), prefers ssDNA over dsDNA and is enzymatically active as a 5'–3' helicase only in NER^31–35^. The CAK module functions to phosphorylate RNA polymerase II (Pol II) and is thus critical for mRNA transcription and processing^36,37^. However, it is not required for NER reaction in vitro^38^ and is shown to dissociate from TFIIH upon UV irradiation in human cells^39^. Structural studies of TFIIH have been a challenge due to its size and its compositional and conformational heterogeneities; available TFIIH structures have so far been limited to a form engaged in transcription initiation together with Pol II in human and in yeast^40–42^ and a more recent structure of human TFIIH in complex with DNA and XPA for NER^43^. Higher-resolution structures of TFIIH including CAK have been determined only for human TFIIH^44,45^. Thus, the structural mechanism of how TFIIH is recruited to lesions by XPC and further propels NER initiation has been a mystery.

Here we determined the structure of yeast TFIIH/Rad4–Rad23–Rad33 bound to DNA containing a single carcinogen-DNA adduct (2-acetylaminofluorene) using cryo-electron microscopy, chemical cross-linking, and mass spectrometry. A ~30 bp stretch of DNA could be mapped along with the protein complex in two distinct segments with respect to the lesion: the dsDNA at the 5' side of the DNA lesion was bound to Ssl2, similarly as observed in the transcription initiation complex, while the duplex at the 3' side of the lesion was bound to the TGD-BHD1 domains of Rad4 near Tfb1 of TFIIH. In between, the DNA was locally (~3 bp) unwound at the lesion, as required for the simultaneous binding with Ssl2 and Rad4. Such structure is also well poised for subsequent NER bubble formation by the translocase activity of Ssl2. The DNA was, however, not yet engaged with Rad3, indicating that the ensuing lesion verification would involve additional steps of conformational changes. Altogether, our study illustrates how the Rad4–Rad23–Rad33 (XPC–RAD23B–CETN2) complex recruits TFIIH to damaged DNA in 3D structural models and provides the groundwork to visualize how the NER initiation is carried out in multiple, coordinated yet distinct steps.

## Results

### Assembly of TFIIH/Rad4–Rad23–Rad33/DNA

**Fig. 1:**
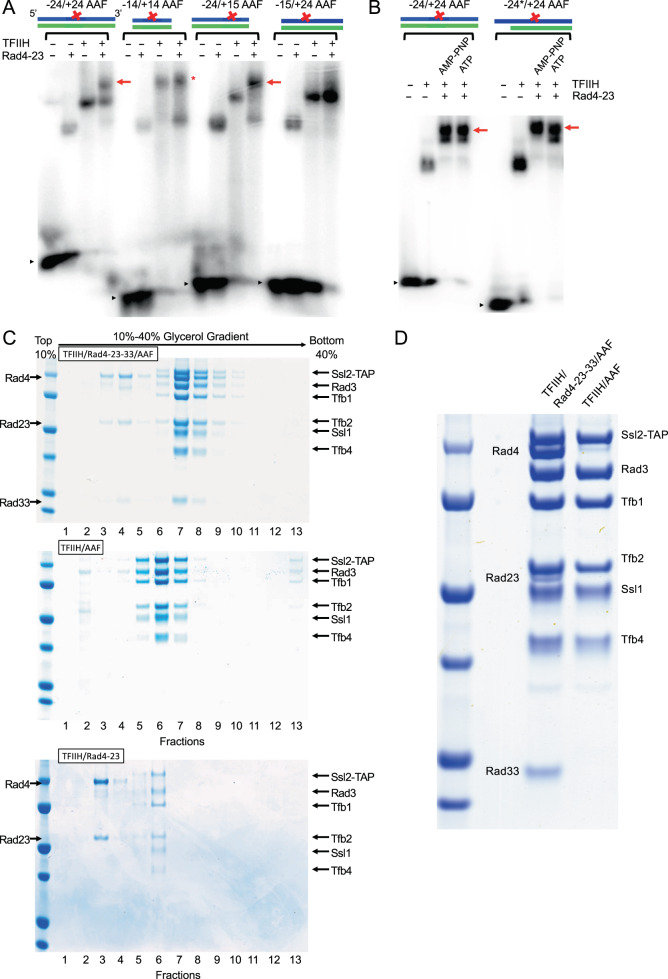
Assembly of coreTFIIH and Rad4–Rad23–Rad33 on AAF-DNA. **A** EMSA of TFIIH and/or Rad4–Rad23 binding to various AAF-modified DNA constructs in the presence of AMP-PNP. Rad4–Rad23 and TFIIH formed complexes with −24/+24 or −24/+15 AAF-DNA (red arrow) but not with −14/+14 or −15/+24 AAF-DNA. Black arrow heads indicate unbound, free AAF-DNA. Asterisk (*) indicates non-stoichiometric TFIIH-DNA complexes lacking Rad4–Rad23. **B** EMSA of TFIIH with Rad4–Rad23 on −24/+24 or −24*/+24 AAF showing the complex formation (red arrow) in the presence of AMP-PNP or ATP. −24*/+24 AAF contains 10-nucleotide (nt) single-stranded overhang on the 5' side of the DNA whereas −24/+24 AAF is fully duplexed. **C** Complex isolation using glycerol gradient sedimentation. CoreTFIIH mixed with −24*/+24 AAF in the presence (top) or absence (middle) of Rad4–Rad23 and Rad33 or coreTFIIH and Rad4–23 without DNA (bottom) were subjected to glycerol gradient centrifugation in the presence of ATP. 24*/+24 AAF was combined with TFIIH, Rad4–Rad23, and Rad33 (top panel) or with TFIIH alone (middle panel), then subjected to glycerol gradient centrifugation in the presence of ATP. SDS-PAGE analysis of the fractions showed that stoichiometric amounts of TFIIH, Rad4–Rad23, and Rad33 were present in a single peak in a slightly higher glycerol density (top panel) than that of the complex with only TFIIH and DNA (middle panel), indicating the formation of a TFIIH/Rad4–Rad23–Rad33/DNA complex. The complex was not formed when DNA was omitted (bottom panel). Note that Rad23 is poorly stained by Coomassie and that sedimentation without DNA was performed at slightly elevated ionic strength (300 mM potassium acetate) to increase the solubility of TFIIH and Rad4–23. Protein standard indicates 100, 75, 50, 37, 25, and 20 kDa (top to bottom). **D** SDS-PAGE of TFIIH/Rad4–23–33/DNA (left) and TFIIH-DNA (right) isolated by gradient sedimentation. Protein standard indicates 100, 75, 50, 37, 25, and 20 kDa (top to bottom).

To assemble a TFIIH/Rad4–Rad23–Rad33/DNA complex (hereafter TFIIH/Rad4–23–33/DNA) suitable for structural study, the 7-subunit coreTFIIH was isolated from yeast as previously published^46^ with slight modifications. Full-length Rad4–Rad23 complex (yeast homologue of XPC–RAD23B) and Rad33 (yeast homologue of CETN2) were obtained by overexpression in insect cells and bacteria, respectively. To identify a suitable DNA substrate for structural studies, we employed gel electrophoresis mobility shift assays (EMSA) with a series of dsDNA containing a 2-acetylaminofluorene adduct (AAF-dG) as an NER lesion (Fig. 1A, B and Supplementary Fig. 1A–C). In addition to a 29-bp DNA fragment (–14 to +14, nucleotide numbering follows the 5'–3'-direction on the damaged strand with respect to the AAF-dG at position +0), which is sufficient for assembly of the Rad4–Rad23/DNA complex^22,23^, we prepared DNA fragments extending ~10 bp on either end (–24/+15, –15/+24), or on both ends (–24/+24). Two DNA constructs including the region from –24 to +15 (–24/+15, –24/+24) supported a stable co-complex formation including both TFIIH and Rad4–Rad23, whereas others (–14/+14 and –15/+24) bound to either TFIIH or Rad4–Rad23 but not to both. The 10-bp dsDNA extension could also be replaced with 10-nt ssDNA without affecting the binding (see –24/+24 vs. –24*/+24, Fig. 1B). The complexes formed in the presence of ATP (4 mM) as well as its non-hydrolyzable analog, AMP-PNP (Fig. 1B). Additionally, the presence of Rad33 did not impair the complex assembly (Supplementary Fig. 1C).

Following these results, we next reconstituted the containing TFIIH, Rad4–23–33, and −24*/+24 AAF-DNA on a preparative scale and sedimented on a 10–40% glycerol gradient in the presence of ATP (Fig. 1C, D). Stoichiometric complexes of TFIIH, Rad4–Rad23, and Rad33 were eluted as a single peak at a slightly higher glycerol density (Fig. 1C top panel, fractions 7–8) than TFIIH alone (Fig. 1C middle panel, fractions 5–7) or free Rad4–Rad23–Rad33 included in a slight excess than TFIIH (Fig. 1C top panel, fractions 3–4). The same homogeneous complex was obtained in the presence of AMP-PNP (Supplementary Fig. 1G) or the three-subunit kinase module TFIIK (Kin28–Ccl1–Tfb3) (Supplementary Fig. 1E). However, it was not possible to assemble the co-complex containing both TFIIH and Rad4–23 when DNA was omitted (bottom panel of Fig. 1C). These results indicate that the stable recruitment of TFIIH to damaged DNA by Rad4 indeed requires DNA in the presence of ATP or AMP-PNP and that such complexation is compatible with holoTFIIH including TFIIK. Additional supporting evidence that the resulting complex represents a stable entity specifically assembled on the DNA, rather than a mixture of non-specifically bound factors, came from factor challenge experiments (Supplementary Fig. 1F–I). When purified Rad2 (yeast homolog of XPG) was added in ~6-fold molar excess over the TFIIH/Rad4–23–33/DNA complex followed by the glycerol gradient sedimentation, ~50% of TFIIH dissociated from Rad4–Rad23–Rad33 and instead bound to Rad2 in the presence of ATP but not AMP-PNP (fractions 8–11 in the bottom versus top panels of Supplementary Fig. 1I). These results corroborate previous biochemical studies with the HeLa nuclear extract, suggesting that XPG fails to stably associate with TFIIH-XPC without ATP and that XPG displaces XPC from TFIIH/DNA when added to TFIIH-XPC in the presence of ATP^47,48^.

### TFIIH/Rad4–Rad23–Rad33 cross-linking mass spectrometry

**Fig. 2:**
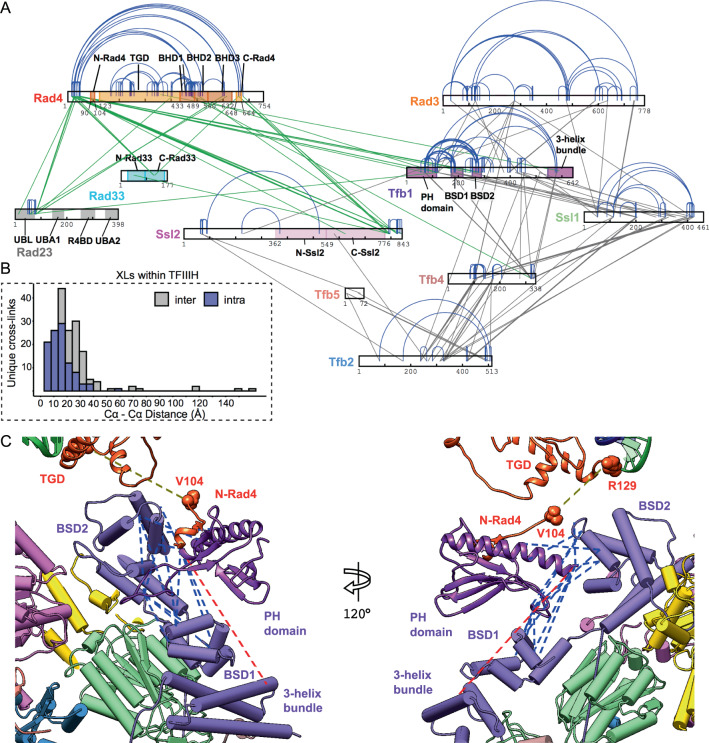
Cross-linking and mass spectrometry of TFIIH/Rad4–Rad23–Rad33/DNA. **A** Non-redundant cross-links identified for TFIIH/Rad4–Rad23–Rad33 on −24/+24 AAF-DNA as a network plot. Intra-subunit cross-links are shown in blue while inter-protein cross-links are shown in gray between TFIIH subunits and in green within Rad4–Rad23–Rad33 and between Rad4–Rad23–Rad33 and TFIIH. **B** Cross-links were validated by mapping them on the atomic model of TFIIH in this study. 93% of cross-links were consistent with the 40 Å upper limit. **C** Current model of the Tfb1 PH domain in TFIIH/Rad4–Rad23–Rad33/DNA complex. Of 16 unique cross-links between the PH domain and the other domains of Tfb1, 15 cross-links had Cα-Cα distances less than 40 Å (blue dotted lines), and 1 cross-link exceed 40 Å (red dotted line).The N-terminal end of the TGD (R129) and the C-terminal end of N-Rad4 (V104) are connected by a green dashed line.

**Fig. 3:**
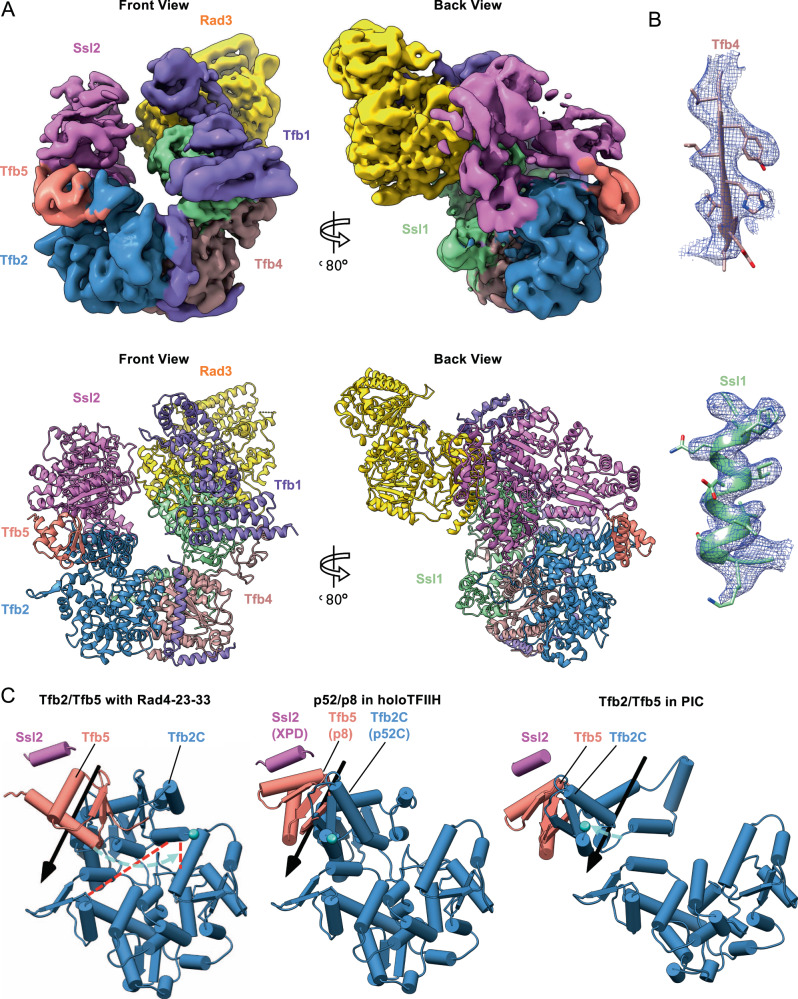
Structure of TFIIH in the TFIIH/Rad4–Rad23–Rad33/DNA complex. **A** Cryo-EM map (Map 1) and corresponding model of TFIIH in NER initiation complex. The map shows clear density for each TFIIH subunit: Ssl2 (pink), Tfb5 (salmon), Tfb2 (blue), Tfb4 (brown), Tfb1 (purple), Ssl1 (green) and Rad3 (gold). Shown are front and 120-degree-rotated back views. **B** EM density maps showing side-chain densities in Tfb4 (top) and Ssl1 (bottom). **C** Conformational changes of Tfb2-Tfb5 upon binding Rad4–Rad23–Rad33/DNA in NER. Relative to human holoTFIIH (PDB:6NMI, middle panel), Tfb2C-Tfb5 undergoes a 60-degree rotation (cyan arrow) hinging at residues 450–452 of Tfb2, accompanied by ~11 Å translation of Ssl2. This contrasts with TFIIH in transcription preinitiation complex (PDB:5OQM, right panel) which undergoes a smaller rotation in the opposite direction (cyan arrows). A cyan sphere depicts the last ordered residue of Tfb2 (p52) and Rad4–23–33 specific cross-links (K171–K495, S80–K506) depicted as dashed red lines.

Further support for the specific assembly of the TFIIH/Rad4–23–33/DNA complex was obtained by cross-linking mass spectrometry (XL-MS) (Fig. 2, Supplementary Dataset). TFIIH/Rad4–Rad23–Rad33 assembled with the –24/+24 AAF-DNA was reacted with MS-cleavable cross-linker disuccinimidyl dibutyric urea (DSBU), and the cross-links were identified by mass spectrometry followed by computational search. A total of 333 cross-links, comprising 257 within TFIIH, 56 within Rad4–Rad23–Rad33, and 20 between TFIIH and Rad4–Rad23–Rad33, were identified. The observed cross-links between TFIIH subunits were in good agreement with those previously obtained from highly purified TFIIH^49–51^. The false-positive discovery rate (FDR) was estimated based on a target-decoy analysis^52^, where decoy was generated by shuffled sequences but with protease sites retained. The FDR was set to 1% to filter each acquisition. FDR was also assessed by comparison to the model of TFIIH obtained at high-resolution from Map 1: Of the 257 cross-links identified here, 182 cross-links could be mapped onto corresponding residues in the model while the other 75 were on unstructured flexible loops and thus were not mapped; 18 cross-links were between residues more than 40 Å apart (the maximum theoretical distance of cross-linking) in the structure of TFIIH, corresponding to a violation rate of 7% (Fig. 2B). Of the 20 cross-links between Rad4–23–33 and TFIIH, 18 cross-links were mapped on the PH domain and BSD2 domain of Tfb1, and the C-terminal region of Ssl2, suggesting that Tfb1 and Ssl2 of TFIIH make the primary contacts with Rad4–Rad23–Rad33. Our results are completely consistent with a prevailing human NER model that the XPC protein interacts with TFIIH through the p62 and XPB subunits, human homologues of Tfb1 and Ssl2, respectively^53,54^, and further extended the model when combined with cryo-EM data (see below). Moreover, intra-subunit cross-links within TFIIH in the TFIIH/Rad4–23–33/DNA complex significantly differed from those in holoTFIIH containing TFIIK (Kin28–Ccl1–Tfb3) (Supplementary Fig. 2A, B). For example, two intra-subunit cross-links within Tfb2 (K171–K495, S80–K506), which were absent in the holoTFIIH are consistent with the rotation of Tfb5/Tfb2C upon binding Rad4–23–33 (XLs indicated by red dashed lines in Fig. 3C). The C-terminal region of Ssl2 (residues 771–843) formed no cross-links with the N-terminal region of Ssl2 (vs. eight cross-links in holoTFIIH) but maintained six intra C-terminal cross-links found in holoTFIIH. The PH domain (residues 1–120) of Tfb1 formed only a total of three cross-links with Tfb2, Tfb4, and Rad3 (vs. two, seven, and nine cross-links in holoTFIIH, respectively). These differences in XL-MS likely reflect changes in the conformation and/or mobility within TFIIH upon binding Rad4–23–33 (Supplementary Fig. 2D and Fig. 3C).

### Cryo-EM structure determination of TFIIH/Rad4–23–33/DNA complex

For cryo-EM studies, coreTFIIH and Rad4–Rad23–Rad33 complexed with –24*/+24 AAF-DNA were prepared by glycerol gradient sedimentation in the presence of glutaraldehyde to enhance sample stability and homogeneity^55^. Aliquots of peak fractions embedded in ice disclosed fields of monodispersed particles in the presence of ATP as well as AMP-PNP (Supplementary Fig. 3A). We imaged ~1 million particles each with ATP or AMP-PNP using Titan Krios electron microscopes equipped with a K3 or K2 direct electron detector. 2D class averaging of particles in the presence of ATP yielded a set of homogeneous classes similar to one another except for differences in direction of view, some of which clearly showed the well-defined features of horseshoe-like TFIIH bound to the Rad4–Rad23–Rad33 complex (left panel of Supplementary Fig. 3B). In contrast, 2D class averages for the AMP-PNP-containing samples were more heterogeneous, hampering structural resolution, although some classes resembled TFIIH (right panel of Supplementary Fig. 3B). Noting the better homogeneity of the ATP-containing complexes, we selected ~250,000 images from this complex through 2D class averaging and subjected them to ab initio calculation of initial maps and subsequent 3D classifications. The resulting maps (Supplementary Movie 1 and Supplementary Table 1) showed clear division in two parts: a well-ordered TFIIH and a flanking disordered Rad4–23–33 (Supplementary Fig. 4). The most populated class resolved TFIIH at a nominal resolution of 3.86 Å, but with disordered density corresponding to the Rad4–Rad23–Rad33 complex bound to DNA (Map 1, EMDB-22587; Supplementary Fig. 4). To better resolve the disordered density, we refined a less populated class containing Rad4–Rad33/DNA at 9.2 Å resolution (Map 2, EMDB-22588; Supplementary Fig. 5A), as well as a class containing Rad4 BHD3 (but not including BHD1-2 and TGD)-Rad33-DNA at 7.9 Å resolution (Map 3, EMDB-22576, Supplementary Fig. 5A). Lastly, multibody refinements^56^ of Map 2 using two masks of coreTFIIH and Rad4–33 improved the resolution of coreTFIIH to 8.1 Å (Supplementary Fig. 5A), allowing us to better describe interactions between TFIIH and DNA. While the conformations of TFIIH in these three maps were similar to one another, they were significantly different from the previous structures of holoTFIIH or TFIIH engaged in transcription^45,57,58^ (Fig. 3C and Supplementary Fig. 2C).

### Structure of TFIIH in the TFIIH/Rad4–Rad23–Rad33/DNA complex

Map 1 resolved TFIIH at a nominal resolution of 3.86 Å, the highest resolution for yeast TFIIH so far reported (Fig. 3A, B, Supplementary Table 1). Local resolution calculations of Map 1 (Supplementary Fig. 3C) indicate that 6 of the 7 subunits of coreTFIIH (Rad3, Tfb1, Tfb2, Tfb4, Tfb5, and Ssl1) were determined at 3–6 Å resolution, whereas Ssl2 was determined at 6–8 Å resolution. Furthermore, although blurred due to the high mobility of the region, a density was also observable, connecting between C-Ssl2 and Tfb1 across the TFIIH complex (Supplementary Fig. 3F). This density is entirely consistent with the position and orientation of Rad4–Rad23–Rad33/DNA as shown in Maps 2 and 3 (further discussed in the next section).

Within TFIIH, the structure of Ssl2 itself was a good match to the previous DNA-bound Ssl2 structures representing the characteristic pre-translocation state of this family of helicases/translocases although ATP could not be resolved in the catalytic site of Ssl2^41–43^. Rad3 and its associated subunit Ssl1 were resolved at the highest resolution (~3–4 Å) within the complex. The DNA-binding channel of Rad3 was occupied with the acidic plug of Tfb1 (residues 337–354) as previously observed in human XPD^45^, indicating auto-inhibition of its DNA-binding.

A yeast homology model of TFIIH was generated using the human cryo-EM structure^45^ as a template for further model building. While the overall subunit organization of TFIIH subunits was similar to that of human TFIIH solved by itself^45^ (~4.6 Å RMSD), a significant difference was found in the position and orientation of Ssl2 and the domain that consists of Tfb5 (residues 2–72) and the C-terminal region of Tfb2 (Tfb2C; residues 412–513)^59^ (Fig. 3C, Supplementary Movie 2). In previous structures of TFIIH, the “open” form of Tfb5-Tfb2C (p52-p8 in humans) was stabilized by hydrophobic interactions between the N-terminal helix of Tfb5 (residues 14–26) and the adjacent hydrophobic helix (residues 563–571) of the Ssl2 C-terminal helicase domain (C-Ssl2, residues 549–776) (middle and right panels of Fig. 3C). In the TFIIH/Rad4–23–33/DNA complex, Tfb5-Tfb2C were rotated ~60° and stabilized in an alternative position (“closed” form) (left panel of Fig. 3C). Compared with the previous structures of TFIIH, this Ssl2-Tfb5-Tfb2 made little contact with the rest of TFIIH, which allowed C-Ssl2 to move away from the rest of TFIIH by ~11 Å (Fig. 3C and Supplementary Fig. 3E). The repositioning of Ssl2 relative to the rest of TFIIH is also consistent with the movement observed after multibody refinement of Map 1 that revealed a ~30° rotation of Ssl2-Tfb5-Tfb2C relative to the rest of TFIIH (Supplementary Fig. 4C–E and Supplementary Movie 4).

Tfb5 stimulates Ssl2’s catalytic activity specifically for the initial NER bubble formation in the presence of Rad4 (XPC)^60^. Altogether, the current structures suggest that the rotation of Tfb5-Tfb2C and thereby unlocking Ssl2-Tfb5-Tfb2C from the rest of TFIIH may be key in stimulating the translocase activity of TFIIH (Ssl2) in an NER-specific manner.

### Structure of Rad4–Rad23–Rad33 in the TFIIH/Rad4–Rad23–Rad33/DNA complex

**Fig. 4:**
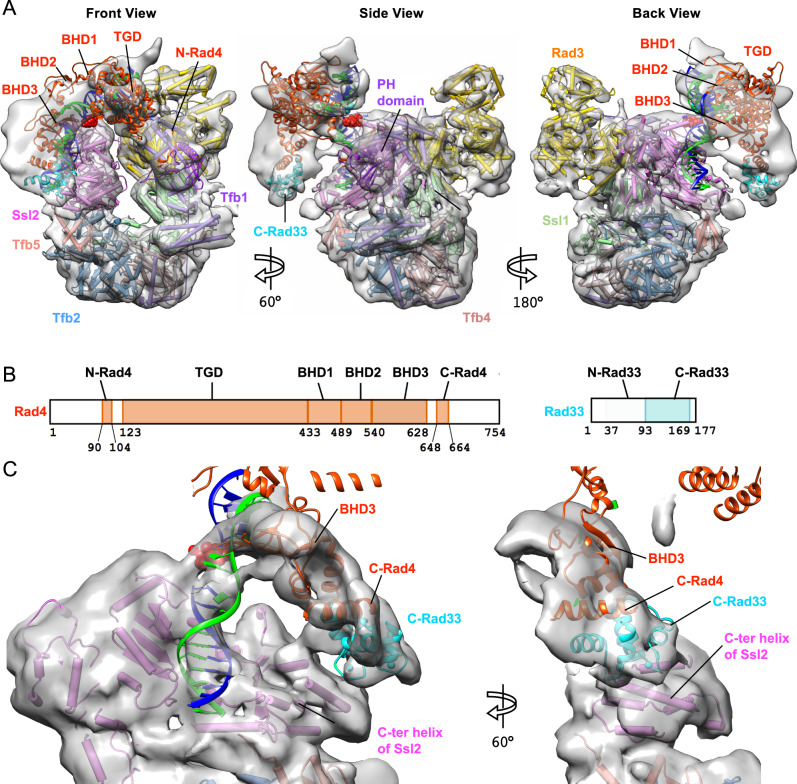
Structure of TFIIH/Rad4–Rad23–Rad33/DNA complex. **A** Composite cryo-EM map of Map 2 and Map 3 of TFIIH with Rad4–Rad23, seen in front (left), side (middle), and back (right) views. The crystal structure of Rad4-DNA complex (PDB:6CFI) was fitted into the density as a rigid body, with the lesion (6–4PP) in red sphere. Rad23 did not show corresponding density. **B** Schematic representation of domains of Rad4 and Rad33. Domains included in the model are colored. **C** 7.9 Å map (Map 3) refined by masking out TGD and BHD1-2. A homology model of Rad33 bound to C-Rad4 (PDB:2GGM) (cyan and orange red) was docked into a corresponding density, guided by cross-links of high confidence between C-Rad4 and the C-terminal region of Ssl2. The N-terminal half of Rad33 was not clearly seen. Seen in the back view.

Compared with Map 1 that resolved TFIIH at high resolution and yet had a poor density for Rad4–Rad23–Rad33, Map 2 clearly showed the elongated density for the Rad4–Rad33 complex bound to DNA at 9.2 Å resolution (Fig. 4A). While the TFIIH structure generally remained the same as in Map 1, there was an additional density adjacent to BSD1 and BSD2 domains of Tfb1 (p62 in human), attributed to the Tfb1 PH domain that is known to interact with the N-terminal region of Rad4/XPC^61,62^. The previous NMR model of the Tfb1 PH domain (residues 1–114) bound to an N-terminal segment of Rad4 preceding TGD (N-Rad4; residues 90–104)^61^ was placed into the density (Fig. 4A), guided by with 9 and 6 cross-links formed with BSD1 and BSD2 of Tfb1, respectively (Fig. 2C). Although the orientation of the PH domain remains tentative due to its limited EM resolution, the position evidently differed from that in transcription^57,58^ (Supplementary Fig. 2C).

**Fig. 5:**
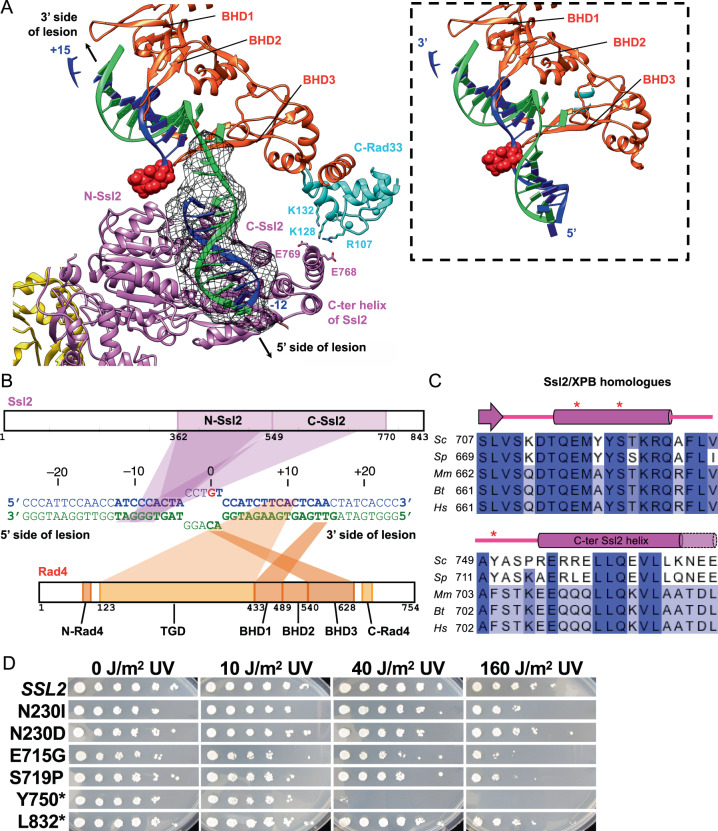
Path of damaged DNA in TFIIH-Rad4/23/33-DNA complex. **A** Zoomed view of protein-DNA interactions at the lesion (red sphere). ~9-bp DNA segment 5' side of the lesion (black mesh) was identifiable on Ssl2 after multibody refinement of Map 2. This segment of dsDNA is untwisted ~150° compared to that in Rad4–Rad23/DNA (PDB:6CFI, inset), enabling simultaneous binding to Rad4 and Ssl2. Map threshold at 0.0013, σ=3.4 with segmentation 4 Å from DNA model. **B** Schematic diagram of protein-DNA interactions suggested by the model. Nucleotide numbering follows the 5'–3'-direction on the damaged strand (blue) with respect to the AAF-dG at position +0. Nucleotides in the model are in bold. **C** Sequence alignment of C- Ssl2 and XPB with secondary structures between *Saccharomyces cerevisiae* (Sc), *Schizosaccharomyces pombe* (Sp), *Mus musculus* (Mm), *Bos taurus* (Bt), and *Homo sapiens* (Hs). Dots indicate positions for mutations of Ssl2 in (D). **D** Mutations of Ssl2 at the interface with Rad33 and Tfb5 confer UV sensitivity. Yeast cultures grown in YPD were diluted and plated. Uncovered plates were then irradiated with UV light (254 nm) at the indicated doses followed by incubation for 3 days. Asterisks indicate C-terminal deletion mutants at indicated positions.

The crystal structure of Rad4–Rad23 bound to a 24-bp double-stranded DNA fragment containing a 6–4 photoproduct UV lesion (PDB:6CFI)^23^ was a good match to the elongated density and was fitted as a rigid body (orange red in Fig. 4A). In the resulting model, the TGD (residues 129–433 of Rad4) domain bound to DNA 3' side of the lesion (mainly at positions +7 to +13) was in close proximity to the Tfb1 PH domain (left panel of Fig. 4A) with the distance between the N-terminal end of the TGD (R129) and the C-terminal end of N-Rad4 (V104) being ~30 Å (green dashed lines in Fig. 2C). On the other hand, the BHD2-3 (residues 489–632 of Rad4) bound to a segment of DNA at the lesion (at positions –1 to +1) were located near Ssl2 (right panel of Fig. 4A). When the DNA double helix 5' side of the lesion^23^ was extended with B-form DNA (Fig. 5), a ~9 bp segment (at positions –12 to –4) was approximately in the position and orientation of DNA path along its binding groove between two ATPase domains of Ssl2^40–43^. The only minor adjustment needed for the modeling was a ~150° rotation (in a direction of unwinding) of the segment of DNA 5' side of the lesion along its axis relative to the 3' side (see next section).

Although the main body of Rad4 (residues 123–632) was in close proximity to the C-terminal helicase domain of Ssl2 (C-Ssl2, residues 549–776), it apparently had little direct contact with C-Ssl2. However, an unassigned EM density bridging C-terminal end of Rad4 BHD3 (residue 632) and C-Ssl2 (Fig. 4A) indicated the presence of Rad33: Rad33 and human centrin 2 (CETN2) are both EF-hand calcium-binding proteins that interact with the conserved C-terminal region of Rad4 and XPC, respectively, and stimulate NER activity^63–69^. To resolve this region, we further refined this map up to ~7.9 Å resolution (Map 3, Fig. 4C and Supplementary Fig. 5A) using a mask that excludes the TGD and BHD1 domains of Rad4. This allowed us to reliably dock a homology model of the Rad33 C-terminal lobe (residues 94–169) bound to the Rad4 C-terminal region (C-Rad4; residues 648–664) into the corresponding density, guided by cross-links of high confidence between C-Rad4 and the C-terminal region of Ssl2 (e.g., Rad4 S660–Ssl2 K796) (Fig. 2A)^70,71^. The N-terminal lobe of Rad33 (residues 26–93) was not observed in the map. The conserved hydrophobic surface of C-Rad4 consisting of Thr651, Ile658, Leu662^71^ pack against hydrophobic residues of BHD3 (Phe585, Leu586, Thr624). The C-terminal helix of C-Ssl2 was extended by one turn (~3 residues) compared to holoTFIIH (PDB:6NMI) so that the acidic patch (Glu768 and Glu769) on its end was in close proximity to basic residues of Rad33 (R107, K128, and K132) (Figs. 5A and 5C). Truncating the C-terminal 125 amino acids of human XPC (corresponding to residues ~617–754 of Rad4) impairs TFIIH binding in vitro^54^. Our model suggests that this is due to the role of C-XPC (Rad4) in binding to CETN2 (Rad33) which in turn directly interacts with the C-terminal helix of XPB (Ssl2). We additionally note that XPA (Rad14 in yeast) also binds this C-terminal helix of XPB (Ssl2)^43^ (Supplementary Fig. 7), and thus may compete with and eventually replace XPC–CETN2 (Rad4–Rad33) from this region of Ssl2 as NER proceeds.

In sum, our model shows that Rad4 binds TFIIH at two points of contact, straddling between the two ends of the horseshoe-shaped TFIIH: N-Rad4 (residues 90–104) contacts with the PH domain of Tfb1, and C-Rad4 (residues 648–664) with the C-terminal helix of C-Ssl2 through Rad33. The main body of Rad4 (residues 123–632 corresponding to TGD and BHD1-3) has no direct contact with TFIIH and is connected to N-Rad4 and C-Rad4 via short flexible linkers, which may account for the lower resolution of the EM maps for Rad4 than for TFIIH.

### Untwisted DNA at the lesion for simultaneous binding to Rad4 and Ssl2

The location of the lesion and the DNA path at its 3' side were reliably deduced from the fitting of the crystal structure of Rad4–Rad23 bound to a 24-bp dsDNA containing a 6–4 photoproduct UV lesion (PDB:6CFI)^23^. Additionally, a ~9-bp double helical segment on the 5' side of the lesion (at positions approximately –12 to –4) was identifiable within the DNA-binding groove of Ssl2 formed between two ATPase domains, in a manner consistent with previous DNA-bound Ssl2/XPB structures^40–43^ (Fig. 5A). Compared with the DNA path in the Rad4–Rad23/DNA crystal structure (PDB:6CFI), this 5'-side DNA segment bound to Ssl2 was rotated by ~150 ° along its axis in a direction of unwinding (left vs right panels in Fig. 5A). This untwisting thus required for the simultaneous binding to Rad4 and Ssl2 on a contiguous DNA, was readily accommodated by the DNA immediately 5' side of the lesion (at positions from approximately –3 to –1), where the DNA had no protein contact and remained flexible and the undamaged strand was barely traceable (Figs. 4C and 5A). This agrees with the previous observation that this 5' side duplex of the DNA lesion was relatively mobile and flexible with respect to the other side of the DNA, bound by Rad4 main body: a hinge point for the flexibility and untwisting was generated at around the lesion (positions 0 to −1), as Rad4 bends and flips out two nucleotide pairs including the damage from the DNA duplex^22,23^. Although in the current model, the DNA untwisting within the TFIIH/Rad4–23–33/DNA complex spans positions ~–3 to ~–1 of the DNA, we note that the exact size and degree of the DNA unwinding could not be determined due to the limited resolution.

### Mutations of Ssl2 adjacent to the interface with Rad33, Tfb5, and Tfb2 confer UV sensitivity

To examine the structure-function relationship in vivo, a subset of novel *ssl2* alleles defective in transcription initiation^72^ were screened for UV sensitivities (Fig. 5C, D). The mutant allele Y750*, a reconstruction of the mutant allele *SSL2-XP*^72,73^ that eliminates 94 C-terminal amino acid residues (residues 750–843), confers extreme UV sensitivity, in good agreement with localization of the binding sites for Rad33 and Rad14 in this region (Fig. 5C, D). Two additional alleles, E715G and S719P, which are located near or at the interface with Tfb5 and Tfb2 (Supplementary Fig. 6), have moderate UV sensitivity. Other ssl2 mutants were either not or were much less sensitive to UV light, suggesting separation of NER and transcription-related functions, or a much greater sensitivity of transcription-related phenotypes than of UV sensitivity. Overall, UV sensitive *ssl2* mutations may impair the binding of NER factors (Rad14, Rad33) or the rotation of Ssl2-Tfb5-Tfb2C (Supplementary Movie 4) required for the structural transition towards unwound state (see Discussion).

## Discussion

Structural and mechanistic studies of NER involving TFIIH have been hampered by difficulties in preparing the relevant NER factors and their assemblies on DNA in sufficient quality and quantity in vitro. With highly purified NER factors, we developed a procedure for isolating homogeneous complexes containing the 7-subunit coreTFIIH and Rad4–Rad23–Rad33 and investigated its assembly on damaged DNA (AAF-dG lesion) for NER initiation. Our structure, as determined by the 3.9–9.2 Å resolution cryo-EM maps and extensive XL-MS analyses, represents the first structure of TFIIH recruited by Rad4(XPC) to damaged DNA. In this structure, ~30-bp stretch of damaged DNA could be visualized with Rad4 on the 3'-side of the lesion and the Ssl2 DNA translocase subunit of TFIIH on the 5'-side, accompanied by ~3–5 bp unwinding and “opening” of the DNA duplex around the lesion. This model agrees with a prevailing model derived from biochemical studies with regard to Rad4(XPC)-TFIIH interactions and positioning of DNA lesion^17,48,53,54,74,75^. Importantly, it provides a key foundation on which the mechanistic intricacies of NER initiation can be understood in 3D molecular structures.

Biochemical studies such as DNA footprinting assays have long been indicated that NER involves progressive DNA unwinding (“opening”) at the lesion as NER factors are recruited to the site in an orderly fashion^47,48^. For various NER lesions, the initial “opening” is first carried out during lesion recognition as Rad4 bends and unwinds DNA around the lesion and flips out two damage-containing nucleotide pairs from the DNA duplex^22,23^. However, how the next level of “opening” and lesion verification involving TFIIH may be executed remained unclear.

### TFIIH loading involves DNA unwinding promoted by Ssl2 and Rad4 simultaneous binding to the DNA

The DNA in our TFIIH/Rad4–23–33/DNA structure shows that the unwinding is extended (positions from –3 to +1) compared to that in Rad4–Rad23/DNA (positions from 0 to +1). The additional unwinding at positions –3 to –1 is a consequence of the simultaneous binding of DNA with Rad4 and Ssl2 (Fig. 5). The relatively small size of unwinding and the absence of Rad3(XPD)-DNA interaction indicate that the structure captures an early step of TFIIH engagement, and that DNA unwinding is needed even for the initial loading of TFIIH. Achieving such simultaneous binding may require ATP hydrolysis: samples assembled without ATP but with AMP-PNP suffered from structural heterogeneity, which could arise from failing to achieve stable dual binding (Supplementary Fig. 3B)^17^.

### NER bubble formation by the joint action of Ssl2 and Rad4

**Fig. 6:**
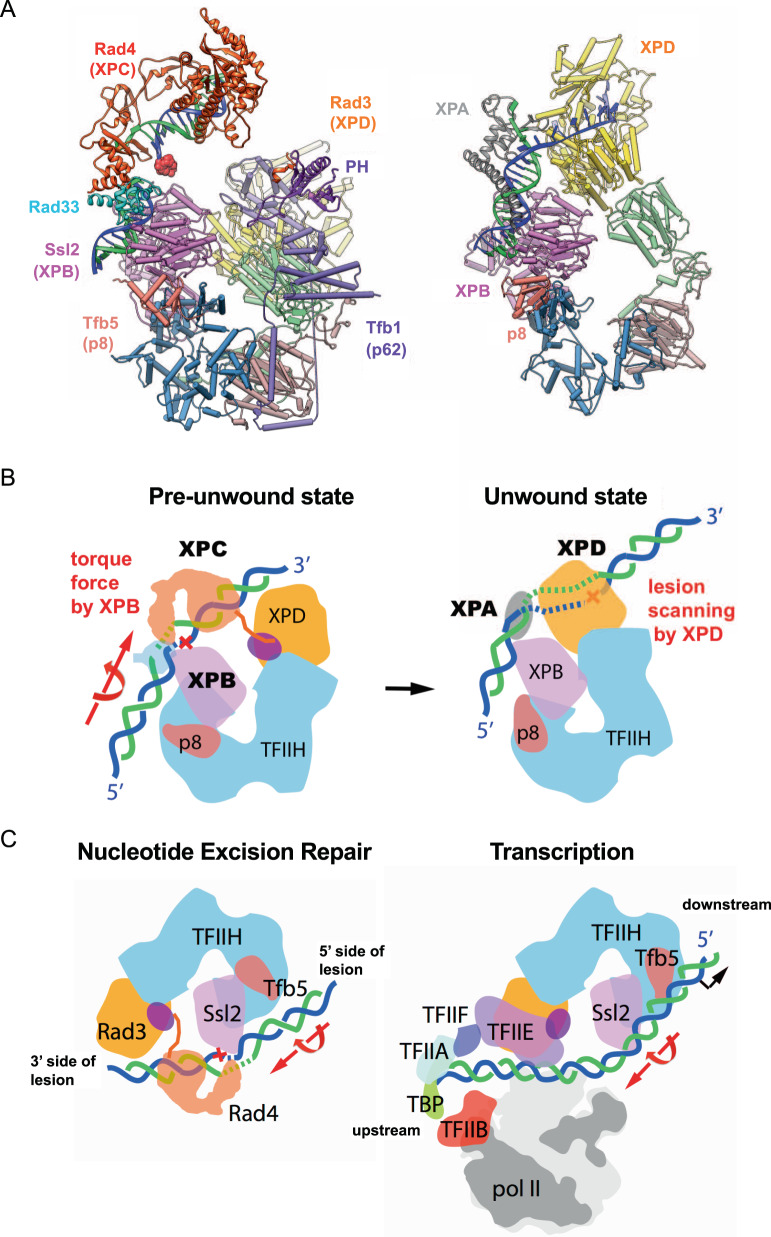
Two structures of TFIIH and schematic model of unwinding of DNA for NER. **A** Ribbon representation of the yeast pre-unwound state (left) and previous structure of the human (artificially) unwound state (right, PDB:6RO4). Two structures are aligned by superimposing Ssl2 and XPB. **B** A model for unwinding damaged DNA by TFIIH, XPC, and XPA in NER. Lesion bound XPC is stabilized on TFIIH primarily through DNA-binding to XPB (left). XPC is then positioned near XPD for lesion scanning and verification by a concerted movement of XPD. The torque force by the translocase activity of XPB opens a bubble near damage, facilitating delivery of the damage DNA to XPD in an unwound form suitable for binding in the DNA-binding cleft (right). This unwinding step is also key to the lesion verification step in which a bulky lesion stalls and blocks the progression of XPD helicase unwinding, thereby allowing the incision complex to be stably formed. **C** A comparison between NER initiation (left) and Pol II transcription initiation (right). Two forms of TFIIH in NER initiation and transcription initiation are aligned. DNA in blue and green is rotated and translocated by Ssl2 in an ATP-dependent manner, in the directions indicated by the large red arrows.

Together with previous biochemical studies, our structure also illustrates how the DNA “opening” may be further extended by Ssl2 for NER initiation, in a manner reminiscent of transcription promoter opening during transcription initiation (Fig. 6C). In RNA polymerase II transcription preinitiation complex, Ssl2 is bound to downstream of the promoter DNA while other factors (e.g., TBP, TFIIF, TFIIE) are bound to the upstream DNA. Like a molecular wrench, the Ssl2 translocase activity on DNA in the presence of the fixed upstream proteins induces DNA melting ~20 bp downstream of the TATA box. This in turn allows the delivery of the template strand to the active center of the polymerase for the transition from a closed to open promoter complex^41,76–79^. In the TFIIH/Rad4–23–33/DNA structure, Ssl2 is bound at the 5' side of the lesion, poised to extend the DNA bubble around the lesion in a manner analogous to how it forms a transcription bubble (Fig. 6C): the NER bubble would extend as Ssl2 reels downstream (5'-side) dsDNA towards Rad4, while Rad4 remains stably bound to upstream (3'-side) DNA around the lesion site (covering positions 0 to +15, Fig. 5B). Consistent with this model, previous permanganate footprinting assays exhibited a permanganate (KMnO_4_) hyperreactive region around positions 0 to –10 on the damaged strand upon full engagement of XPB^48^. Along with Ssl2’s translocase activity, Rad4 is likely to be essential in the NER bubble formation. As previously noted, the position and orientation of Rad4 with respect to TFIIH is held in place by its dual interaction with Ssl2 and Tfb1 of TFIIH through its flexible C- and N-termini, respectively. Without Rad4 holding the 3' side of the DNA as an anchor, the DNA may freely rotate around its helical axis, thereby failing to convert the torsional stress generated by Ssl2 translocation into DNA unwinding. Rad4 BHD3 that wedges between two DNA strands at the lesion may also play a key role in keeping the two strands apart, stabilizing ssDNA conformations generated during the bubble extension.

### Structural transition towards Rad3-engaged “unwound” state

Once XPC recruits TFIIH to the damaged site, XPD is supposed to scan the damaged strand by its 5'–3' helicase activity in order to verify a bona fide bulky NER lesion and prevent futile excision of non-bulky, undamaged DNA. Recently, a cryo-EM structure mimicking the Rad3-engaged “unwound” state was reported, where coreTFIIH and XPA was assembled on a fork DNA substrate containing unduplexed, single-stranded DNA segment that permitted TFIIH and XPA loading while bypassing the requirement for XPC (Fig. 6A, B, and Supplementary Fig. 6)^43^.

Compared with TFIIH structures solved as a part of the transcription preinitiation complex or as holoTFIIH by itself, XPD(Rad3) in the “unwound” structure moved ~80 Å to bind to the ssDNA segment in its DNA-binding channel, whereas XPB kept the same position while bound to a dsDNA segment. When our yeast “pre-unwound” structure was aligned with the human “unwound” structure by superposing Ssl2 on XPB, the ~80 Å movement of XPD(Rad3) from the pre-unwound to unwound states did not cause a major steric clash with Rad4. The repositioned XPD was also suitably positioned to bind to the damaged DNA strand in its DNA-binding cleft (with 5′ to 3′ ssDNA-binding polarity from left to right in Fig. 6A, B). This suggests that the structural transition from “pre-unwound” to “unwound” state may happen while retaining Rad4 (Supplementary Movie 3).

By contrast, the same superposition showed that Rad33 and XPA(Rad14) would encounter steric clash with each other as both bind to the C-terminal helix of C-Ssl2 (Supplementary Fig. 7). This suggests that Rad33 and Rad14 are mutually exclusive when they bind to Ssl2. Biochemical studies have suggested that Rad33 and Rad14 functions at distinct steps in NER: the former in promoting DNA damage recognition with XPC(Rad4) and early TFIIH loading^62,63^ and the latter in activating XPD(Rad3) helicase after initial TFIIH loading by XPC(Rad4)^17,28,43^.

Thus, the transition between the “pre-unwound” and “unwound” stages of the NER initiation may entail the binding of Rad14 to TFIIH-DNA which may displace Rad33 from Ssl2 but still retain Rad4–23–33 on the assembly through the binding between N-Rad4 and the Tfb1 PH domain. The ultimate release of Rad4 from the NER assembly would take place when XPG(Rad2) competes with XPC(Rad4) in binding with p62(Tfb1)^47,61,80–82^.

Finally, the multibody refinement of TFIIH provided an additional clue regarding the structural transition from the “pre-unwound” to “unwound” states: the striking ~30 Å movement of Ssl2-Tfb5-Tfb2C directed towards Rad3, brings the damaged strand into close proximity to the ssDNA-binding cleft of Rad3 (Supplementary Movie 4). Such movement may represent a transition state on the path to a stable “unwound” state. In our samples, however, such transition may have been present only transiently, easily reverting back to the “pre-unwound” state; complete and stable conversion into the “unwound” state may require additional factors such as Rad14(XPA) and RPA^83–85^ accompanied by the large movement of Rad3/XPD as described above^43^. Also, a longer DNA segment on the 5' side of the lesion than our current design may be required in order to permit a larger bubble formation.

### The role of CAK in NER initiation

CAK(TFIIK), as a part of holoTFIIH, is essential for transcription initiation but not for NER, at least in vitro^28,38,60^, and the presence of CAK may be inhibitory to DNA-binding as well as helicase/ATPase activities associated with XPB and XPD^28,86,87^. Nevertheless, holoTFIIH rather than coreTFIIH is probably recruited to NER in cells^39,88^, and failure in the removal of CAK has been linked with failure in NER^39^. However, CAK likely does not dissociate immediately after TFIIH’s recruitment by XPC(Rad4), as observed by the co-complexation of TFIIK-containing holoTFIIH with Rad4–Rad23–Rad33/DNA in yeast (Supplementary Fig. 1E) as well as in humans^28^. The full detachment of CAK from the NER assembly is reported to involve the XPA C-terminus^39^. These observations point to the importance of considering how CAK(TFIIK) associates within the NER assembly and how its dissociation is executed in a controlled fashion.

Although our study does not directly answer these questions, it provides a novel structural hypothesis that can be further tested. In the structure of holoTFIIH^45^, a long α-helix of the MAT1(Tfb3) subunit of CAK(TFIIK) engages with coreTFIIH via two anchors, one on XPB(Ssl2) and the other on XPD(Rad3). In our structure, we observed greater variability in the position of Ssl2 than in XPB in the human holoTFIIH^45^ as well as increased flexibility of the Rad3 Arch domain compared with the XPD Arch domain in human holoTFIIH^45^. These indicate that the CAK(TFIIK) bridging between XPB and XPD may restrict the conformational freedom of coreTFIIH required for the structural transition towards the unwound state. We posit that the repositioning of XPB(Ssl2) relative to the rest of TFIIH upon binding XPC(Rad4)/DNA as observed here (Fig. 3C, Supplementary Fig. 3E) could facilitate CAK(TFIIK) detachment from its anchor on XPB(Ssl2) while retaining the other anchor on XPD(Rad3). This may relieve the structural constraint imposed by CAK at least partially and help deliver the damage DNA to XPD (e.g., by activating the translocase activity of XPB(Ssl2) and enabling the rotation of Ssl2-Tfb5-Tfb2C directed towards Rad3 (Supplementary Movie 4)). The complete release of CAK(TFIIK) may then be achieved in subsequent NER steps, such as recruitment of XPA, XPG, and RPA^39^. The increased flexibility of the XPD Arch domain, achieved as CAK(TFIIK) fully detaches, may be critical in the ssDNA-binding and scanning function of the XPD(Rad3) helicase^86,87^.

Together, our study sets the stage for detailed mechanistic investigations on NER initiation and its subsequent steps. For instance, how does the translocase activity of XPB and helicase activity of XPD in TFIIH orchestrate DNA unwinding and subsequent lesion scanning for damage verification^17,28^? How does XPA and RPA stimulate this process^28,89^? Our structural study opens new doors to the structural studies of the entire NER machinery at a bona fide NER substrate and develop novel therapeutic interventions against various NER-linked diseases.

## Methods

### Protein purification

The Rad4–Rad23 and complexes were prepared as previously described^22^. Briefly, the Hi5 insect cells co-expressing the Rad4–Rad23 complex were harvested 2 days after infection. After lysis, the proteins were purified using His-Select Nickel agarose resin (Sigma) and anion exchange chromatography (Source Q, GE Healthcare), followed by thrombin digestion and cation exchange (Source S, GE Healthcare) and gel-filtration (Superdex200, GE Healthcare) chromatography. The final sample was concentrated by ultrafiltration to ∼13 mg/ml in 5 mM bis–tris propane–HCl (BTP-HCl), 800 mM sodium chloride, 5 mM dithiothreitol (DTT), pH 6.8.

Holo- and coreTFIIH were purified from yeast as previously described^29^ with minor modifications. In short, tfb6Δ strain containing TAP tags on TFIIH subunits Tfb4 (holoTFIIH) or Tfb4 and Ssl2 (coreTFIIH, Supplementary Table 2) was grown by fermenter (Eppendorf) in 100 L of YPAD medium to OD 10.0. The whole-cell lysate was prepared by bead beating in Buffer A (50 mM HEPES pH 7.6, 1 mM EDTA, 5% glycerol, 400 mM potassium acetate, 2-mercaptoethanol, and protease inhibitors). Following the addition of 100 mM ammonium sulfate and 0.12% PEI, lysed cells were stirred for 1 h and centrifuged, and then the cleared lysate was loaded onto an IgG column. The column was washed with 5–10 column volumes of buffer 300 (50 mM HEPES pH 7.6, 1 mM EDTA, 5% glycerol, 300 mM potassium acetate, 2 mM DTT, and protease inhibitors) then resuspended in buffer 300 and allowed to settle overnight. IgG beads were washed by batch with another 10 column volumes of buffer 300 over the course of 24 h. TFIIH was treated with TEV for 15 h in buffer 300, eluted from the IgG column, spin concentrated then loaded onto an UnoQ column (Bio-Rad). TFIIH was eluted by a salt gradient of concentration from 300 mM to 1.2 M potassium acetate. Fractions containing 10-component holoTFIIH, six-component coreTFIIH, and five-component TFIIH lacking Ssl2 were concentrated separately, aliquoted, and flash frozen.

Rad33 was overexpressed in bacteria and purified by the following method. Rosetta(DE3) (Fisher Scientific) were transformed with pETDuet-RAD33 plasmid and grow overnight. 2 L of Terrific Broth (TB) was inoculated with overnight culture and grown to OD600 = 1.0. Cells were induced with 1 mM Isopropyl β-D-1-thiogalactopyranoside (IPTG) for 4 h and harvested by centrifugation (Sorvall LYNX 6000, ThermoFisher Scientific) at 13,000 x*g* for 10 min. Cells were resuspended in lysis buffer (50 mM HEPES (pH 7.6), 500 mM sodium chloride, 5 mM 2-mercaptaethanol, 20 mM imidazole, 5% (v/v) glycerol and protease inhibitors) and lysed by sonication. Lysate was clarified by centrifugation at 30,000 x*g* for 45 min and loaded onto a HisTrap column (GE Healthcare) equilibrated in nickel buffer A (50 mM HEPES (pH 7.6), 500 mM sodium chloride, 5 mM 2-mercaptaethanol, 20 mM imidazole, 5% (v/v) glycerol). The column was washed with 10 column volumes of 3% nickel buffer B (50 mM HEPES (pH 7.6), 500 mM sodium chloride, 5 mM 2-mercaptaethanol, 750 mM imidazole, 5% (v/v) glycerol) and then eluted with a linear gradient of nickel buffer A to nickel buffer B over 90 mL. Peak fractions were pooled, diluted ~5 fold with buffer 0 (20 mM HEPES, 2 mM DTT), and loaded onto a Q column (GE Healthcare). Rad33 was then eluted from the Q column using a linear gradient of Q buffer A (20 mM HEPES, 100 mM sodium chloride, 2 mM DTT, 5% (v/v) glycerol) to Q buffer B (20 mM HEPES, 1 M sodium chloride, 2 mM DTT, 5% (v/v) glycerol). Peak fractions were pooled, buffer exchanged to buffer 300 (20 mM HEPES, 300 mM potassium acetate, 5 mM DTT) during concentration, and frozen.

Rad2 was cloned into a His Sumo vector (pGVD52-RAD2HisSumo, Supplementary Table 2), overexpressed in bacteria and purified by the following method. Rosetta(DE3) (Fisher Scientific) were transformed pGVD52-RAD2HisSumo. Transformed cells were grown in TB media at 37 °C, induced with 0.4 mM IPTG at OD600 0.9, and grown overnight at 16 °C. Cells were harvested by centrifugation (Sorvall LYNX 6000, ThermoFisher Scientific) at 13,000x*g* for 10 min and resuspended in a lysis buffer that contained 50 mM Tris-HCl (pH 7.5), 500 mM sodium chloride, 40 mM imidazole, 5% (v/v) glycerol, and 5 mM 2-mercaptoethanol and protease inhibitors. Cells were lysed by sonication and lysate was clarified by centrifugation at 30,000 x*g* for 45 min. The supernatant was loaded on a HisTrap column (GE Healthcare) equilibrated in lysis buffer. The column was then washed with lysis buffer that contained 1.5 M sodium chloride and then buffer A (50 mM Tris-HCl (pH 7.5), 500 mM sodium chloride, 40 mM imidazole, 5% (v/v) glycerol, 5 mM 2-mercaptoethanol) and then eluted from the column with a linear gradient of nickel buffer A to nickel buffer B over 90 mL. 6xHis-Ulp1 protease was added to the protein-containing fractions, which were then dialyzed overnight in a buffer that contained 20 mM Tris-HCl, 250 mM sodium chloride, 5% (v/v) glycerol, 5 mM 2-mercaptoethanol. Dialyzed samples were re-applied on a HisTrap column, and protein that contained flow-through was loaded onto a Heparin HP column (GE Healthcare). Rad2 was eluted from by a linear gradient of heparin buffer A (20 mM Tris-HCl, 250 mM potassium acetate, 5% (v/v) glycerol and 2 mM DTT) to heparin buffer B (20 mM Tris-HCl 1 M potassium acetate, 5% (v/v) glycerol and 2 mM DTT). Peak fractions were pooled and concentrated.

Rad14 was cloned into a pET His vector (Supplementary Table 2), overexpressed in bacteria, and purified by the following method. Rosetta(DE3) (Fisher Scientific) were transformed pET-RAD14His. Transformed cells were grown in TB media supplemented with 10 μM zinc chloride at 37 °C, induced with 1 mM IPTG at OD600 1.0, and grown for 3  h at 25 °C. Cells were harvested by centrifugation (Sorvall LYNX 6000, ThermoFisher Scientific) at 13,000 x*g* for 10 min and resuspended in a lysis buffer that contained 100 mM Tris-HCl (pH 8.0), 500 mM sodium chloride, 20 mM imidazole, 10% (v/v) glycerol, and 2 mM DTT, 10 μM zinc chloride and protease inhibitors. Cells were lysed by sonication and lysate was clarified by centrifugation at 30,000 x*g* for 45 min. The supernatant was loaded on a HisTrap column (GE Healthcare) equilibrated in nickel buffer A (100 mM Tris-HCl (pH 7.5), 100 mM sodium chloride, 20 mM imidazole, 5% (v/v) glycerol) and eluted from the column with a linear gradient of nickel buffer A to nickel buffer B (buffer A + 500 mM imidazole) over 90 mL. Protein-containing fractions were then loaded onto a Q HP column (GE Healthcare) and eluted from by a linear gradient of Q buffer A (100 mM Tris (pH 7.5), 100 mM sodium chloride, 5% (v/v) glycerol and 5 mM DTT) to Q buffer B (100 mM Tris (pH 7.5), 100 mM sodium chloride, 5% (v/v) glycerol and 5 mM DTT). Peak fractions were dialyzed into buffer 300 (20 mM HEPES (pH 7.6), 200 mM potassium acetate, 5% glycerol, 1 mM DTT), concentrated and frozen.

### N-AAF DNA synthesis and purification

N-acetoxyacetomino fluorinated DNA adducts (Supplementary Table 2) were purified via a modified protocol from the previous described^16^. In all,1.25 nmol of oligonucleotides with single G residues were incubated with 0.1 mM N-acetoxy-2-acetylaminofluorene in TE buffer at 37 °C for 3 h in a light sensitive container. The top AAF-modified oligonucleotides were separated from unmodified ones by C18-reversed phase high-performance liquid chromatography (Beckman Ultrasphere 5 μm; φ4.6 × 250 mm). The column was an equilibrated sample loaded with a mixture of 100 mM triethylamine acetate (Sigma) and acetonitrile (95:5). Bound oligonucleotides were eluted by linearly increasing the acetonitrile concentration from 10 up to 30%. Complementary bottom unmodified oligonucleotides were purified by Urea-PAGE. Top and bottom oligos were mixed, annealed, and then the double-stranded damaged or undamaged product was purified by size exclusion chromatography (Superose 6, GE Healthcare) in buffer 300 (20 mM HEPES pH 7.6, 300 mM potassium acetate, 5 mM DTT).

### Electromobility shift assays

Purified DNA substrates were labeled by mixing 15 pmol of AAF DNA, with T4 PNK (New England BioLabs), PNK buffer, and P^32^ gamma-ATP for 90 min at 37 °C. Excess P^32^ gamma-ATP was then separated from labeled AAF DNA by G50 spin column (GE Healthcare). Labeled AAF DNA was mixed 1:4 with unlabeled AAF DNA prior to use for electromobility shift assays (EMSA).

EMSAs were performed by mixing equal concentrations (250 nM) of Rad4–Rad23, coreTFIIH, and labeled/unlabeled AAF DNA in buffer 100 (20 mM HEPES, 100 mM potassium acetate, 2 mM magnesium acetate, 5 mM DTT) with additional factors as indicated. Reactions were incubated at room temperature for 20 min. Reactions were run on 0.2x TBE agarose gels at 4 °C for 2 h. Gels were vacuum-dried (BioRad) and imaged by a phosphoimager.

### Rad2/Rad14 competition assays

To prepare samples for competition assays, 120 pmol of Rad4–Rad23, 120 pmol of Rad33, and 120 pmol of −24/+24 AAF were mixed and dialyzed to assembly buffer (20 mM HEPES pH 7.6, 300 mM potassium acetate, 2 mM magnesium acetate, 5% (v/v) glycerol, 5 mM DTT) for 4 h. Dialyzed Rad4–Rad23–Rad33/AAF was then added to 0.83 nmol of coreTFIIH with 5 mM ATP or 2 mM AMP-PNP 2 mM magnesium acetate and 120 pmol of Rad14 or Rad2. Reactions were incubated for 60 min. TFIIH/Rad4–Rad23–Rad33/AAF + /− Rad14/Rad2 were then sedimented at 52,000 rpm for 6 h and 35 min in a 10 to 40% glycerol gradient (20 mM HEPES pH 7.6, 150 mM potassium acetate, 2 mM magnesium acetate, 4 mM DTT, 2 mM ATP or 1 mM AMP-PNP) at 4 °C using a Beckman SW 60 Ti rotor. Glycerol gradients were prepared using a Gradient Master device (BioComp Instruments). Samples were then fractionated with a Piston Gradient Fractionator (BioComp Instruments). Protein distribution was analyzed by TCA precipitation of fractions followed by SDS-PAGE analysis. Gel images were scanned and then densitometry analysis was performed in ImageJ^90^ and data were plotted in Prism (GraphPad). Data points and raw gels provided in the Source Data file.

### Cryo-EM sample preparation and data collection

To prepare samples for cryo-EM analysis, 1.15 nmol of Rad4–Rad23, 1.18 nmol of Rad33, and 1.15 nmol of −24*/+24 AAF were mixed and dialyzed to assembly buffer (20 mM HEPES, 300 mM potassium acetate, 2 mM magnesium acetate, 5% (v/v) glycerol, 5 mM DTT) for 4 h. Dialyzed Rad4–Rad23–Rad33/AAF was then added to 0.83 nmol of coreTFIIH, with 5 mM ATP and 2 mM magnesium acetate, and incubated for 60 min. TFIIH/Rad4–Rad23–Rad33/AAF complex was then prepared as previously described^55^: samples were sedimented at 52,000 rpm for 6 h and 35 min in a 10 to 40% glycerol gradient (20 mM HEPES, 150 mM potassium acetate, 2 mM magnesium acetate, 4 mM DTT, 2 mM ATP) at 4 °C using a Beckman SW 60 Ti rotor. Glycerol gradients were prepared using a Gradient Master device (BioComp Instruments). When cross-linking was used for EM analysis, 0.125% (v/v) glutaraldehyde was added to the 40% glycerol solution before gradient preparation. Samples were then fractionated with a Piston Gradient Fractionator (BioComp Instruments). Cross-linking reactions were quenched by the addition of glycine-HCl buffer (pH 7.5) to a final concentration of 40 mM. The peak was analyzed by TCA precipitation of uncrosslinked fractions followed by SDS-PAGE analysis. Peak crosslinked fractions were concentrated and then frozen. A similar procedure was followed for preparation of AMP-PNP state of TFIIH/Rad4–Rad23–Rad33/AAF complex by replacing ATP with AMP-PNP in all of the steps.

To prepare cryo-EM grids, purified TFIIH/Rad4–Rad23–Rad33/AAF was thawed and dialyzed in EM buffer (20 mM HEPES pH 7.6, 150 mM potassium acetate, 5 mM DTT) for 30 min immediately prior to making grids. Two microliters of the dialyzed sample was then applied to glow-discharged (1 min, easiGlow, Pelco) R2/2 200- or 300-mesh Quantifoil holey carbon grids (Electron Microscopy Sciences). The grids were subsequently blotted for 2 or 3 sec, respectively, using Whatman Grade 41 filter paper (Sigma-Aldrich) and flash-frozen in liquid ethane with a Leica EM CPC manual plunger (Leica Microsystems). EM grids were prepared in batches and the freezing conditions were optimized by screening on a FEI TF20 microscope operating at 200 kV and equipped with a FEI Falcon III direct electron detection camera at the Electron Microscopy Research Lab (University of Pennsylvania).

Cryo-EM specimens were imaged at two different settings. Dataset 1 was collected at University of Massachusetts Medical School was collected using a FEI Titan Krios G3i transmission electron microscope operating at 300 kV, equipped with a K3 direct electron detector (Gatan) and a Bioquantum energy quantum filter (Gatan). Data was collected by SerialEM^91^ by image shift and at a nominal magnification of 105,000x in super-resolution mode (pixel size of 0.415 Å) at a defocus range between 0.8 and 2.8 μm. A total of 6234 images were over the course of one and a half days. Exposures were 2.6 sec with movies divided into 30 frames at a targeted nominal dose of 50 e^−^/Å2.

Dataset 2 was collected at the NIH National Center CryoEM Access and Training (NCCAT) at the New York Structural Biology Center (NYSBC) using a FEI Titan Krios transmission electron microscope operating at 300 kV, equipped with a K2 Summit direct electron detector (Gatan) and a Bioquantum energy quantum filter (Gatan). Data was collected by Appion^92^ by image shift and at a nominal magnification of 13,000x in super-resolution mode (pixel size of 0.504 Å) at a defocus range between 0.8 and 2.8 μm. A total of 2073 images were over the course of 2 days. The exposure time was 5 s at a nominal dose of 42 e^−^/Å2.

### Image processing and 3D reconstruction

A combination of software including cryoSPARC v2.12.4^93^, Relion 3.0.8^94^, and Relion 3.1 were used to process both cryo-EM datasets. Datasets were processed independently as follows. The K3 dataset (Umass) dataset was motion-corrected with MotionCorr2^95^ then imported into cryoSPARC for CTF correction with CTFFIND4^96^. A total of 1,159,898 particles were picked by blob picking, and two rounds of reference-free 2D classification were performed to remove particles that lacked clear features (Supplementary Fig. 3B), resulting in a subset of 250,223 particles. This subset was then subjected to initial model calculation followed by four rounds of heterogenous refinement then non-uniform refinement all in cryoSPARC. The resulting map (7.1 Å resolution) showed clear TFIIH density with noticeable rotation of Tfb2C-Tfb5. This map was low pass filtered and then used as an initial model for 2 rounds of heterogeneous refinement of the entire 1,159,898 particle dataset which resulted in four maps with reasonable TFIIH features.

The best map, containing 196,883 particles, yielded a 4.9 Å resolution map after non-uniform refinement in cryoSPARC. These particles were then transferred to Relion 3.0.8 for an additional round of 3D classification. The best class contained 56,101 particles and yielded a 4.8 Å resolution map after 3D auto-refinement in Relion 3.0.8. After multiple rounds of CTF refinement and Bayesian polishing a 3.86 Å resolution map was obtained (Map 1).

A similar method as described above was used for processing the K2 (NCCAT dataset). The K2 dataset (NCCAT) dataset were motion-corrected with MotionCorr2^95^ then imported into cryoSPARC for CTF correction with CTFFIND4^96^. A total of 595,544 particles were picked by blob picking, and a single round of reference-free 2D classification were performed to remove particles that lacked clear features, resulting in a subset of 155,529 particles (subset 1). These particles were imported into Relion 3.1 and subjected to 3D classification without a mask (Supplementary Fig. 4B). One of the class contained 63,672 particles with clear density for both Rad4 and TFIIH. Auto-refinement of this map using a soft mask containing the entire Rad4 and TFIIH yielded a map of 9.25 Å resolution (Map 2). To obtain a higher-resolution of Rad4 BHD3 and TFIIH, another subset of 73,146 particles (subset 2) from cryoSPARC was imported into Relion 3.1 and subjected to 3D classification with a soft-edge mask containing Rad4 BHD3 and TFIIH. Auto-refinement of the best resolved class containing 32,858 particles using the same soft-edge mask yielded a map of 7.95 Å resolution (Map 3).

Multibody refinement of Map 1 was conducted by making independent masks of Ssl2-Tfb5-Tfb2C and Rad3/Ss1/Tfb1/Tfb4/Tfb2N (Supplementary Fig. 5C). The first three eigenvectors accounted for 55% of total motion in the reconstruction (Supplementary Fig. 4D). Eigenvectors one and three represent small local motions but eigenvector two represented a significant constant motion of Ssl2-Tfb2-Tfb5 (Supplementary Movie 4, Supplementary Fig. 4E). Multibody refinement of Maps 2 and 3 was conducted by making independent masks of coreTFIIH and Rad4–23–33 (Supplementary Fig. 5A). Multibody refinement resulted in significantly improved density of coreTFIIH bound DNA (8.1 Å Supplementary Fig. 5B, 7.0 Å Supplementary Fig. 5C respectively). Composite maps were constructed in UCSF Chimera^97^ using the vop maximum command.

### Model building and refinement

To build the model of TFIIH in the high-resolution map (Map 1), we started with rigid body fitting of homology models^98^ of TFIIH subunits using the EM structure of human TFIIH (PDB:6NMI) as a template^45^ using UCSF Chimera^97^. Tfb5-Tfb2C (residues 438–508) were well outside the density and fitted into the density as a rigid body in UCSF Chimera. The resulting model for seven yeast TFIIH subunits was then used as a template for structure refinement using RosettaCM^99^ and iterative refinements with Coot (Emsley and Cowtan, 2004) and Phenix1.16^100^. The final model statistics are found in Supplementary Table 1.

For the TFIIH/Rad4–Rad23–Rad33/AAF model, the TFIIH model obtained from Map 1 was docked into the map containing density for Rad4–Rad33/AAF (Map 2). Then a crystallographic model of a Rad4–Rad23 bound to a 6–4 photoproduct UV lesion (PDB:6CFI) was docked as a rigid body into the EM map (Map 2) without any deviations. DNA 3' side of the lesion (0 to +15) was retained as in the crystal, while the DNA 5' side of the lesion was removed. Rad23 was not included in the model due to the lack of the corresponding density. DNA 5' side of the lesion (–12 to –4) was modeled using that on XPB (PDB:6RO4) as a template. A homology model of Rad33 bound to C-Rad4 was generated using the crystal structure of human Centrin 2 bound to XPC peptide (PDB:2GGM) as a template^70^, and was fitted into the corresponding density as a rigid body^97^. The final model containing Rad4, Rad33, TFIIH, and DNA was subjected to energy minimization and rigid body refinement against Map 3 with Phenix1.16^100^. PH domain bound to N-Rad4 (PDB:2M14) was placed in the corresponding density and refined as a rigid body with Phenix1.16^100^. The final (only most plausible) model was chosen based on the fitting score. Figures were made using a combination of UCSF Chimera^97^ and UCSF ChimeraX^101^.

### Cross-linking mass spectrometry sample preparation

One hundred fifty micrograms of TFIIH/Rad4–Rad23–Rad33/AAF was assembled by the same method for EM analysis but was subjected to XL-MS analysis without isolation by glycerol gradient sedimentation. Assembled TFIIH/Rad4–Rad23–Rad33/AAF was diluted to a concentration of 1 mg/mL with buffer 300 (20 mM HEPES (pH = 7.6), 300 mM potassium acetate, 5% glycerol, 5 mM DTT) and then mixed with 50 mM (final 6 mM) disuccinimidyl dibutyric urea (DSBU) (Thermo Fisher Scientific). Cross-linking reaction was incubated on ice for 2 h and then was quenched by adding 1 M (final 50 mM) of ammonium bicarbonate, and then further stopped by TCA precipitation. Crosslinked proteins were precipitated with 20% (w/v) trichloroacetic acid (TCA, Sigma) on ice for 90 min. Proteins were pelleted by centrifugation at 21,000x*g* for 15 min and washed with 10% TCA in 100 mM Tris-HCl and then with acetone (Fisher Scientific). The solvent was decanted, the pellet air-dried, and then stored at −80 °C for analysis by mass spectrometry.

Crosslinked proteins were resuspended in 50 μl of resuspension buffer (2.5% SDS and 50 mM triethylammonium bicarbonate (TEAB) final concentrations) and reduced with final 10 mM DTT (US Biological) for 30 min at 30 °C, followed by alkylation with final 50 mM iodoacetamide (Sigma Aldrich) for 30 min at 30 °C. The proteins were captured in an S-Trap^TM^ mini column (Protifi, C02-mini) to remove contaminants, salts, and detergents and concentrate the proteins in the column for efficient digestion then digested with trypsin (Thermo Fisher Scientific) in 1:10 (w/w) enzyme/protein ratio for 1 h at 47 °C. Peptides eluted from this column, with 50 mM TEAB, 0.2% formic acid, and 60% acetonitrile in order, were vacuum-dried and resuspended with the peptide fractionation buffer [(70% (v/v) LC-MS grade water (Thermo Fisher Scientific), 30% (v/v) acetonitrile (ACN, Thermo Fisher Scientific) and 0.1% (v/v) trifluoroacetic acid (TFA, Thermo Fisher Scientific)]. To enrich for crosslinked peptides, peptides were first fractionated using an AKTA Pure 25 with a Superdex 30 Increase 3.2/300 column (GE Life Science). System was flowed at a rate of 30 μL/min of fractionation buffer and 100 μL fractions were collected from the beginning of the sample-loop injection to 1.5 column volumes. Based on the elution profile, fractions containing enriched crosslinked peptides of higher molecular masses were vacuum-dried and resuspended in 0.1% (v/v) TFA in LC-MS grade water for mass spectrometry analysis.

Fractions were analyzed by a Q-Exactive HF mass spectrometer (Thermo Fisher Scientific) coupled to a Dionex Ultimate 3000 UHPLC system (Thermo Fischer Scientific) equipped with an in-house made 15 cm long fused silica capillary column (75 μm ID), packed with reversed phase Repro-Sil Pure C18-AQ 2.4 μm resin (Dr. Maisch GmbH, Ammerbuch, Germany). Elution was performed by the following method: a linear gradient from 5 to 45% buffer B (90 min), followed by 90% buffer B (5 min), and re-equilibration from 90 to 5% buffer B (5 min) with a flow rate of 400 nL/min (buffer A: 0.1% formic acid in water; buffer B: 80% acetonitrile with 0.1% formic acid). Data were acquired in data-dependent MS/MS mode. Full scan MS settings were as follows: mass range 300–1800 m/z, resolution 120,000; MS1 AGC target 1E6; MS1 Maximum IT 200. MS/MS settings were: resolution 30,000; AGC target 2E5; MS2 Maximum IT 300 ms; fragmentation was enforced by higher-energy collisional dissociation with stepped collision energy of 25, 27, 30; loop count top 12; isolation window 1.5; fixed first mass 130; MS2 Minimum AGC target 800; charge exclusion: unassigned, 1, 2, 3, 8 and >8; peptide match off; exclude isotope on; dynamic exclusion 45 sec. Raw files were converted to mgf format with TurboRawToMGF 2.0.8^102^.

### Crosslinked peptide search

To identify and validate crosslinked peptides the search engine MeroX 2.0.1.4^103^ was used with a custom curated FASTA file that contains NER subunits. MeroX was run in RISEUP mode, with default cross-linker mass and fragmentation parameters for DSBU and the following adjusted parameters: precursor mass range, 1000–10,000 Da; minimum precursor charge 4; precursor and fragment ion precisions 5.0 and 10.0 ppm, respectively; the maximum number of missed cleavages 3; carbamidomethylation of cysteine and oxidation of methionine, as fixed and variable modifications, respectively. The common Repository of Adventitious Proteins (cRAP) FASTA database was appended to the search by MeroX to account for common contaminants in proteomics experiments. Results were filtered for score (>10) and false discovery rate, FDR ( < 1%). The in-house R script was utilized to automate the execution of MeroX to submit to a cluster, combine the results, prioritize K-K pair if lysine miscleavage presents and localization score ties, and filter the results to show only unique pairs with the best scores. The Xlink Analyzer plug-in^104^ for UCSF Chimera was used for visualization of the cross-links and placement of the Tfb1 PH domain.

### *ssl2* phenotypic analysis

A subset of novel *ssl2* alleles defective in transcription initiation and TSS selection^72^ were screened for UV sensitivity. These alleles were identified based on randomized mutagenesis of *SSL2* and screening for phenotypes we have found to correlate with initiation defects, sensitivity to mycophenolic acid (MPA), and activation of a transcriptional fusion of *HIS3* and the *IMD2* promoter. These phenotypes are previously described and relate to shifting of TSSs upstream (MPA sensitivity) or downstream (activation of *IMD2::HIS3* reporter)(see Malik et al^105^ for the description of phenotypes and how Pol II mutants with these phenotypes show altered TSS usage). See Qiu et al^106^ illustrating how Pol II mutants with these noted phenotypes alter TSS usage genome-wide. *ssl2* alleles have been screened similarly (preprint is forthcoming) and their phenotypes are along lines of classical *ssl2* alleles examined in Goel et al^107^ for transcription defects. For UV sensitivity testing, liquid cultures of relevant strains were grown overnight in YPD. 10-fold serial dilution series of each strain were spotted onto minimal medium lacking leucine (SC-Leu) and exposed to indicated doses of 265 nm UV light using an FB-UVXL-1000 UV cross-linker (Fisher). Plates were incubated at 30˚C in the dark for 3 days and then imaged. Yeast media were as described previously^108^ with modifications previously described^109^. Sequence alignments were generated by clustal omega multi sequence alignment and colored by sequence conservation in Jalview^110^.

### Statistics and reproducibility

Electromobility shift assays and gradient assembly assays were performed at least twice unless otherwise specified with representative results shown. Experiments for Fig. 1C bottom panel and Supplementary Fig. 1E were performed once.

### Reporting summary

Further information on research design is available in the Nature Research Reporting Summary linked to this article.

## Supplementary information

Supplementary Information

Description of Additional Supplementary Files

Supplementary Data 1

Supplementary Movie 1

Supplementary Movie 2

Supplementary Movie 3

Supplementary Movie 4

Reporting summary

## Data Availability

Cryo-EM maps and models were deposited in the Electron Microscopy Data Bank (EMDB-22587 for Map 1, EMDB-22588 for Map 2, EMDB-22576 for Map 3). The atomic coordinates were deposited in the Protein Data Bank (accession code: 7K01, 7K04, 7M2U). Cross-linking Mass Spectrometry data of TFIIH/Rad4–Rad23–Rad33/AAF complex was deposited in the PRIDE repository under accession number PXD021212. Source data for data underlying Fig. 1 and Supplementary Fig. 1 are provided with this paper. Other data are available from the corresponding authors upon reasonable request. Source data are provided with this paper.

## Source data

Source Data

## Code availability

The custom R script for automation of MeroX analysis by cluster and compilation of results is currently still under development but is available by request by contacting the corresponding author KM (kenjim@pennmedicine.upenn.edu).
