# Supplementary material for: Cryo-EM structure of TFIIH/Rad4–Rad23–Rad33 in damaged DNA opening in nucleotide excision repair
Source: Nat Commun. 2021 Jun 7;12:3338. doi: 10.1038/s41467-021-23684-x (PMC8184850; doi:10.1038/s41467-021-23684-x)
Supplement: Supplementary file 1 — Supplementary Information [file 41467_2021_23684_MOESM1_ESM.pdf]

Supplementary Information for Cryo-EM structure of TFIIH/Rad4-Rad23-Rad33 in damaged DNA opening in Nucleotide Excision Repair

Trevor van Eeuwen[1,2,3], Yoonjung Shim[4], Hee Jong Kim[1,2,3,5], Tingting Zhao[6], Shrabani Basu[6], Benjamin A. Garcia[1,5], Craig Kaplan[6], Jung-Hyun Min[4*], and Kenji Murakami[1,3,*]

[1] Department of Biochemistry and Biophysics, Perelman School of Medicine, University of Pennsylvania, Philadelphia, PA 19104, USA.

[2] Biochemistry and Molecular Biophysics Graduate Group, Perelman School of Medicine, University of Pennsylvania, Philadelphia, Pennsylvania 19104, USA

[3] Penn Center for Genome Integrity, Perelman School of Medicine, University of Pennsylvania, Philadelphia, PA 19104, USA.

[4] Department of Chemistry and Biochemistry, Baylor University, Waco, Texas 76798, USA.

[5] Epigenetics Institute, Department of Biochemistry and Biophysics, Perelman School of Medicine, University of Pennsylvania, Philadelphia, Pennsylvania 19104, USA.

[6] Department of Biological Sciences, University of Pittsburgh, Pittsburgh, Pennsylvania 15260, USA.

*Correspondence to: kenjim@pennmedicine.upenn.edu; JungHyun_Min@baylor.edu

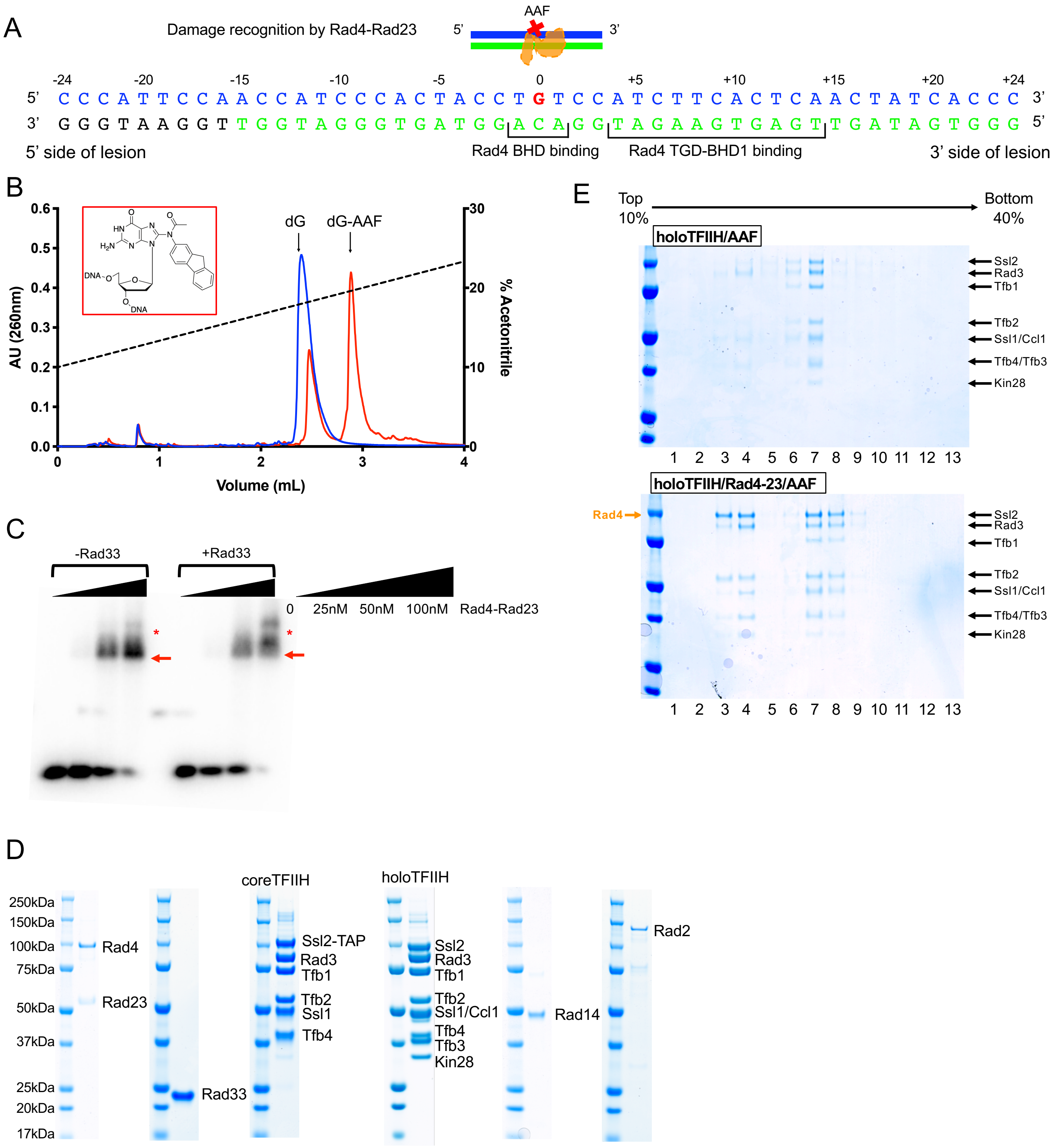
A
Damage recognition by Rad4-Rad23
AAF
5' C C C A T T C C A A C C A T C C C A C T A C C T G T C C A T C T T C A C T C A A C T A T C A C C C 3'
3' G G G T A A G G T T G G T A G G G T G A T G G A C A G G T A G A A G T G A G T T G A T A G T G G G 5'
5' side of lesion
Rad4 BHD binding
Rad4 TGD-BHD1 binding
3' side of lesion
B
AU (260nm)
% Acetonitrile
Volume (mL)
dG
dG-AAF
C
-Rad33
+Rad33
0 25nM 50nM 100nM Rad4-Rad23
D
coreTFIIH
holoTFIIH
250kDa
150kDa
100kDa
75kDa
50kDa
37kDa
25kDa
20kDa
17kDa
Rad4
Rad23
Rad33
Ssl2-TAP
Rad3
Tfb1
Tfb2
Ssl1
Tfb4
Ssl2
Rad3
Tfb1
Tfb2
Ssl1/Ccl1
Tfb4
Tfb3
Kin28
Rad14
Rad2
E
Top 10%
Bottom 40%
holoTFIIH/AAF
Ssl2
Rad3
Tfb1
Tfb2
Ssl1/Ccl1
Tfb4/Tfb3
Kin28
1 2 3 4 5 6 7 8 9 10 11 12 13
holoTFIIH/Rad4-23/AAF
Rad4
Ssl2
Rad3
Tfb1
Tfb2
Ssl1/Ccl1
Tfb4/Tfb3
Kin28
1 2 3 4 5 6 7 8 9 10 11 12 13

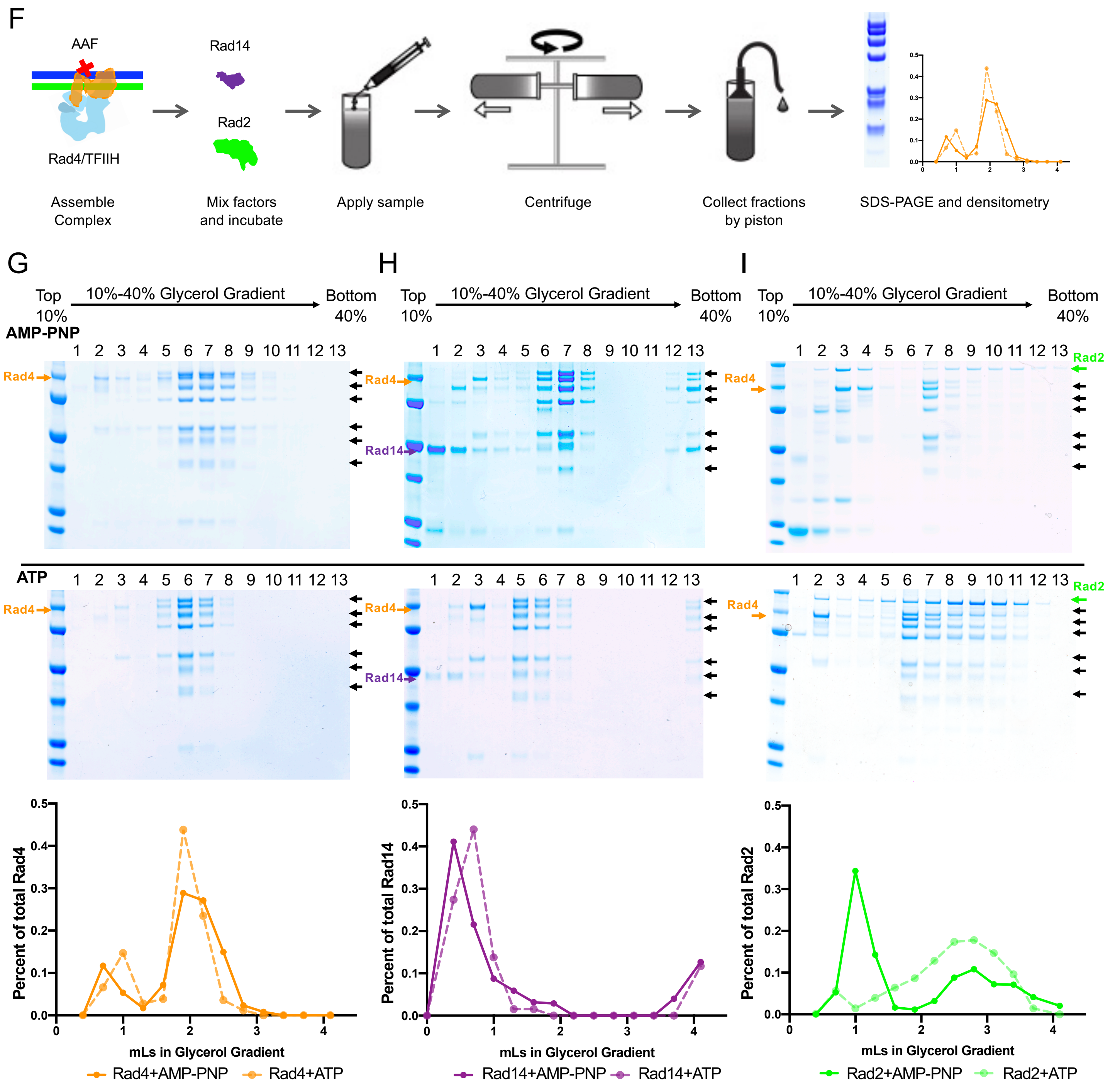

Supplementary Figure 1.
(A) Schematic illustration of Rad4 bound to a lesion (AAF)-containing DNA duplex (upper panel) and the sequence of the -24/+24 AAF-DNA used as a DNA template in this study (lower panel). AAF-dG is in red at position 0. **(B)** Separation of AAF-dG oligonucleotide (red) from single stranded dG oligo (blue) by HPLC reverse phase chromatography. N-Acetoxy-2-acetamidofluorene reacted specifically at guanosine (inset) under conditions described in Methods. **(C)** EMSA of TFIIH with increasing amounts (0-100 nM) of Rad4-Rad23 with -24/+24 DNA in the presence or absence of 100 nM of Rad33. Stoichiometrically bound complexes are indicated by red arrows, whereas some non-specific/non-stoichiometric complexes are indicated by red asterisks. Represe **(D)** SDS-PAGE analysis of purified NER proteins. **(E)** Same as in Figure 1C but with holoTFIIH containing TFIIK (Ccl1, Tfb3 and Kin28). The holoTFIIH/Rad4-23/AAF (lower panel) sedimented faster than holoTFIIH/AAF (upper panel). Rad4 comigrated with Ssl2 on SDS-PAGE with increased intensity of the band. **(F)** Schematic illustration of competitive binding assay with Rad14 and Rad2. Figure adapted from previous work [1,2]. **(G)** Isolation of the TFIIH/Rad4-Rad23-Rad33 complex with -24*/+24 AAF in the presence of adenylyl-imidodiphosphate (AMP-PNP, top) or adenosine triphosphate (ATP, middle). **(H-I)** The TFIIH/Rad4-

23-33/DNA complex was combined by addition of 6-fold molar excess of Rad14 (H) or Rad2 (I) and then subjected to glycerol gradient sedimentation in the presence of AMP-PNP (top) or ATP (middle). Proteins were analyzed by SDS-PAGE analysis. Proteins of interest (Rad4, Rad14 or Rad2) were quantified by densitometry (bottom). TFIIH-Rad4/23/33-DNA complex was stable during sedimentation in the presence of AMP-PNP or ATP in fractions 6-8 (G). Rad14 failed to bind TFIIH/Rad4-23-33/DNA complex in either AMP-PNP or ATP (H). Rad2 did not show robust binding with AMP-PNP (I, top) but did bind with ATP, and facilitated dissociation of Rad4-Rad23 (I, middle). Protein standard indicates 100, 75, 50, 37, 25, 20kDa (top to bottom) (G,H) and 150, 100, 75, 50, 37, 25, 20kDa (top to bottom) (I).

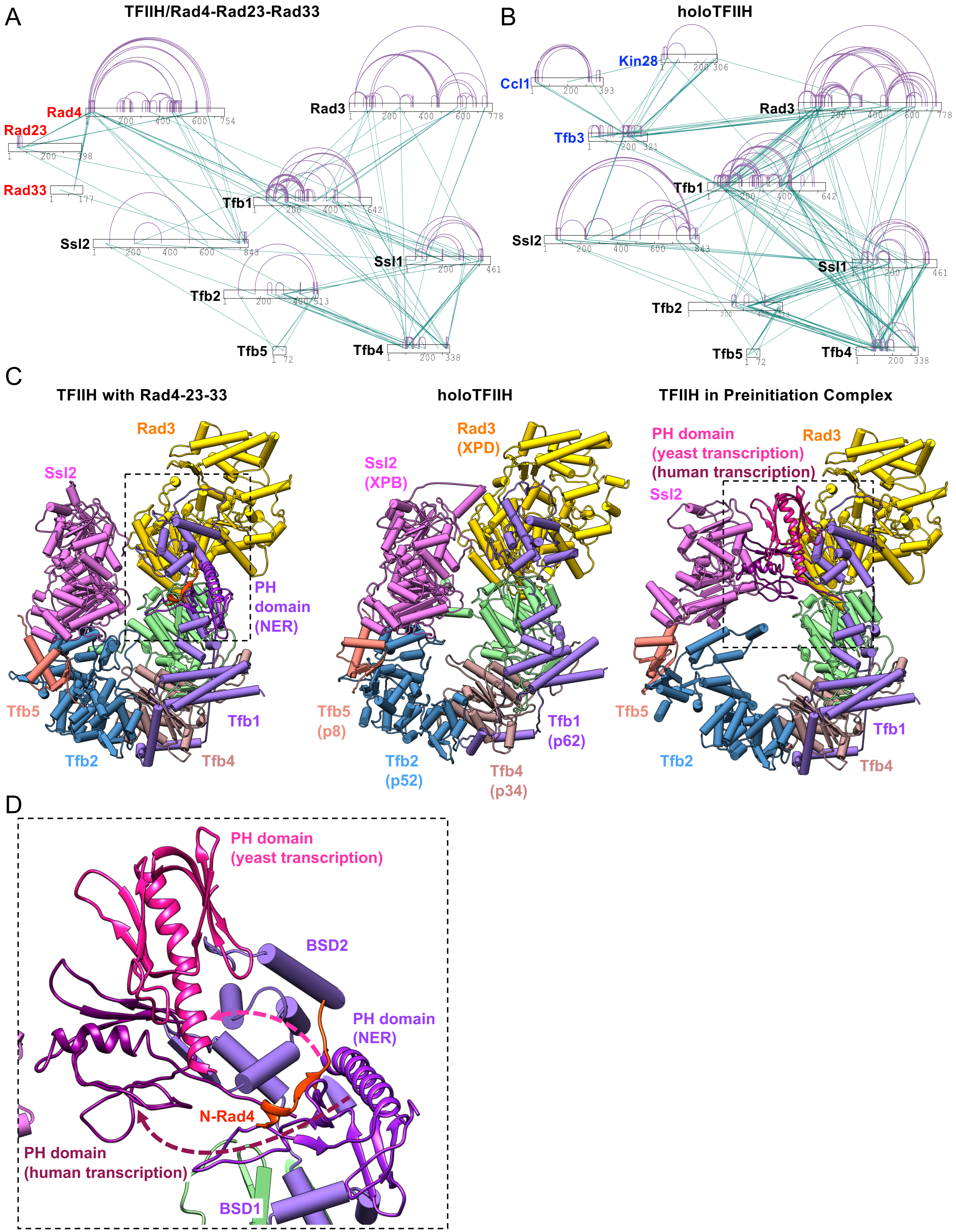

Supplementary Figure 2.

(A-B) Cross-links identified for TFIIH/Rad4-23-33/DNA (A) and holoTFIIH containing TFIIK (B) as a network plot. Intra-molecular cross-links are in purple while inter-molecular cross-links are in green. Control

experiments of holoTFIIH were performed under same experimental conditions. Details of XL-MS of holoTFIIH were previously described [3]. **(C)** Comparison of coreTFIIH structures in the context of TFIIH/Rad4-23-33/DNA (left, current study), holoTFIIH (middle, PDB:6NMI), and the yeast and human transcription preinitiation complexes (right, PDB:5OQJ, 6ORL). **(D)** Comparison of Tfb1 PH domain positioning in transcription initiation (yeast PIC magenta, PDB:5OQJ; human PIC maroon, PDB:6ORL) and NER initiation (purple). Structures aligned by superposition of the Tfb1 BSD and the 3-helix bundle domains.

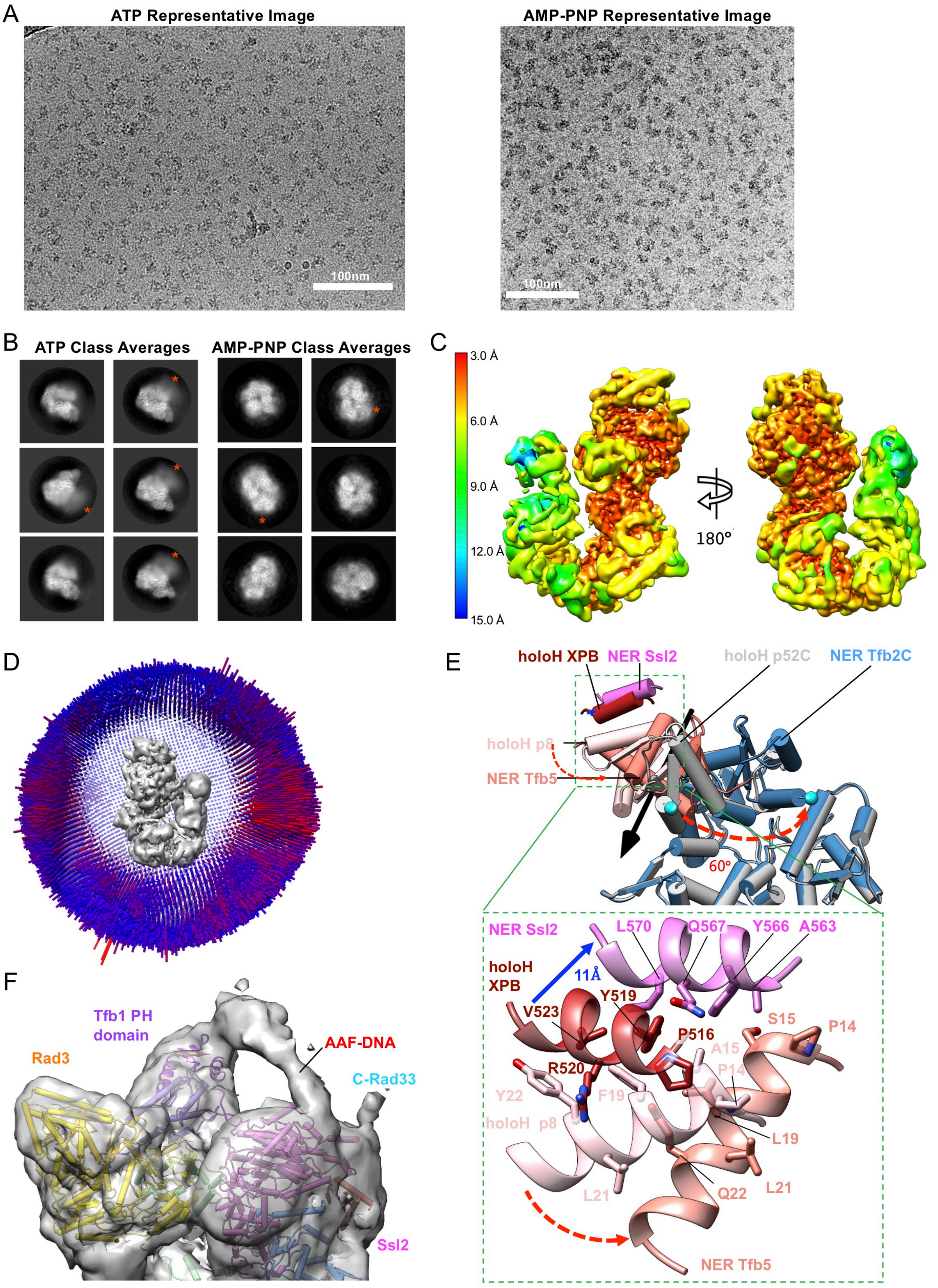
A
ATP Representative Image
100nm
AMP-PNP Representative Image
100nm
B
ATP Class Averages
AMP-PNP Class Averages
C
3.0 Å
6.0 Å
9.0 Å
12.0 Å
15.0 Å
180°
D
E
holoH XPB
NER Ssl2
holoH p52C
NER Tfb2C
holoH p8
NER Tfb5
60°
NER Ssl2
L570
Q567
Y566
A563
holoH XPB
11Å
Y519
V523
P516
S15
P14
A15
P14
R520
Y22
F19
holoH p8
L19
L21
Q22
L21
NER Tfb5
F
Tfb1 PH domain
AAF-DNA
Rad3
C-Rad33
Ssl2

Supplementary Figure 3.
(A) Representative electron micrograph of TFIIH/Rad4-23-33/DNA complex assembled in the presence of adenosine triphosphate (ATP) or adenylyl-imidodiphosphate (AMP-PNP), a non-hydrolysable analogue. Representative image of 6234 images for ATP dataset and 2073 images for AMP-PNP dataset. Scale bar represents 100nm. **(B)** Representative 2D class averages obtained from reference-free 2D classification of TFIIH/Rad4-23-33/DNA complex by Relion 3.0. Rad4 (red asterisk) in the samples assembled in the presence of ATP (left) or AMP-PNP (right) shows alternative binding locations relative to TFIIH. **(C)** Local resolution of the coreTFIIH within the ternary complex in the ATP state as calculated by Blocres at FSC=0.5. Ssl1, Tfb4 and Rad3 represent a high-resolution core of the complex with local resolution ~3-4 angstroms. Ssl2 and Tfb2C-Tfb5 formed a structural module variable in position and orientation relative the rest of TFIIH with local resolution ~7-9 angstroms. Map colored by local resolution from 3.0 angstroms (red) to 15.0 angstroms (blue) as indicated by scale bar (left). **(D)** Euler plot distribution of particle views in the final reconstruction, where the view representation increases from blue to red. **(E)** Conformational changes of Tfb2-Tfb5 upon binding Rad4-Rad23-Rad33. The structure of human TFIIH solved by itself (PDB:6NMI) was aligned by superposing p52 on its homolog Tfb2 in the TFIIH/Rad4-23-33/DNA complex. Compared with the human TFIIH structure, Tfb2C-Tfb5 in our complex undergoes a 60° rotation hinging at residues 450-452 of Tfb2, accompanied by ~11 Å translation of Ssl2 (inset). Cyan spheres depict the last ordered residues of Tfb2 or p52. *(Inset)* Enlarged view of the interactions between the N-terminal helix of Tfb5 (salmon) and the hydrophobic helix of C-Ssl2 (pink) along with its human counterparts, p8 (light pink) and XPB (brown red). **(F)** Density attributable to Rad4, Rad33 and AAF-DNA is visible at a lower contour level in Map 1 as well.

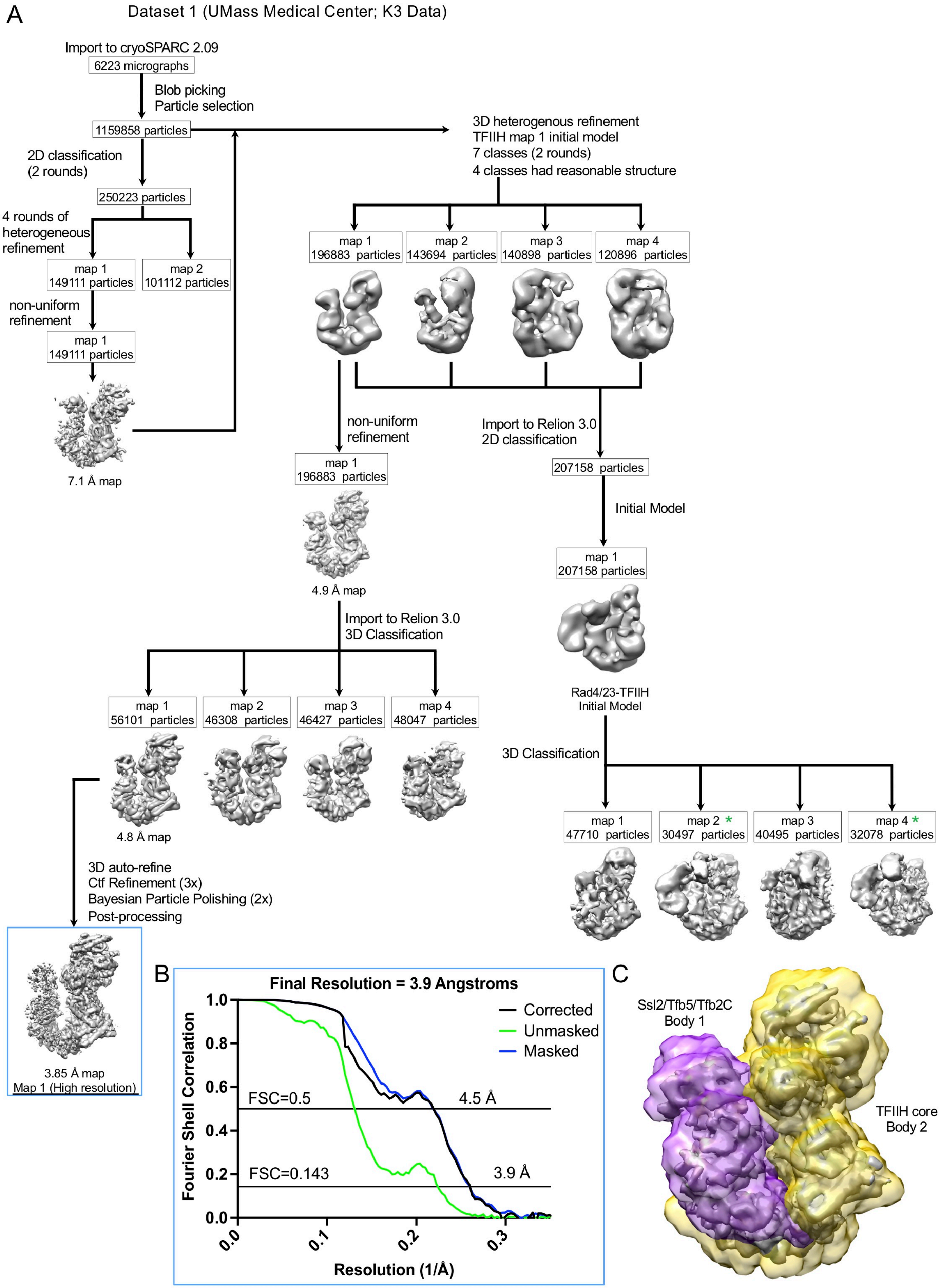
A
Dataset 1 (UMass Medical Center; K3 Data)
Import to cryoSPARC 2.09
6223 micrographs
Blob picking
Particle selection
1159858 particles
2D classification
(2 rounds)
250223 particles
4 rounds of
heterogeneous
refinement
map 1
149111 particles
map 2
101112 particles
non-uniform
refinement
map 1
149111 particles
7.1 Å map
3D heterogenous refinement
TFIIH map 1 initial model
7 classes (2 rounds)
4 classes had reasonable structure
map 1
196883 particles
map 2
143694 particles
map 3
140898 particles
map 4
120896 particles
non-uniform
refinement
map 1
196883 particles
4.9 Å map
Import to Relion 3.0
2D classification
207158 particles
Initial Model
map 1
207158 particles
Rad4/23-TFIIH
Initial Model
Import to Relion 3.0
3D Classification
map 1
56101 particles
map 2
46308 particles
map 3
46427 particles
map 4
48047 particles
4.8 Å map
3D Classification
map 1
47710 particles
map 2 *
30497 particles
map 3
40495 particles
map 4 *
32078 particles
3D auto-refine
Ctf Refinement (3x)
Bayesian Particle Polishing (2x)
Post-processing
3.85 Å map
Map 1 (High resolution)
B
Final Resolution = 3.9 Angstroms
Fourier Shell Correlation
Resolution (1/Å)
Corrected
Unmasked
Masked
FSC=0.5
4.5 Å
FSC=0.143
3.9 Å
1.0
0.8
0.6
0.4
0.2
0.0
0.0
0.1
0.2
0.3
C
Ssl2/Tfb5/Tfb2C
Body 1
TFIIH core
Body 2

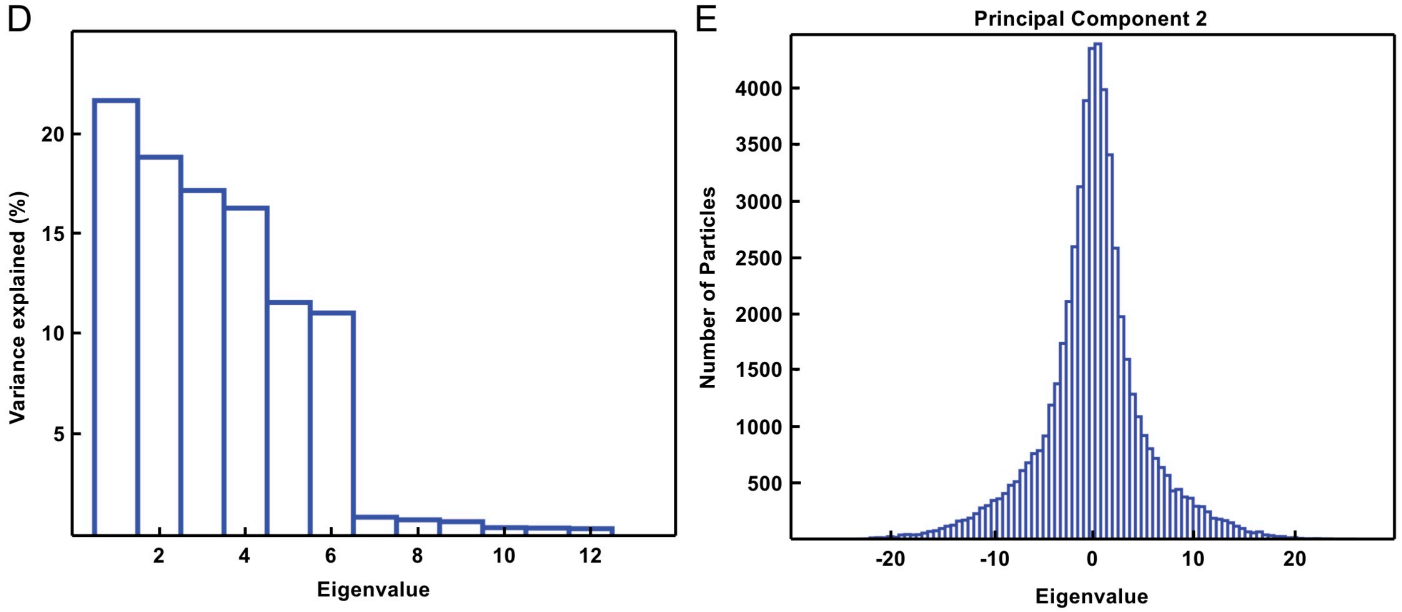

Supplementary Figure 4.
(A) Summary of data processing procedures and workflow of TFIIH/Rad4-23-33/DNA complex with ATP from K3 data collection. In short, data was motion corrected by MotionCorr2 and imported to cryoSPARC for CTF correction, particle picking and 2D classification. An initial reconstruction showed clear and novel TFIIH conformation at 4.9 angstroms. Import into Relion 3.0, followed by further 3D classification followed by CTF refinement, Bayesian polishing, and post processing yielded a reconstruction of 3.9 angstroms. **(B)** Fourier shell correlation (FSC) curves by postprocessing in high resolution TFIIH map (Map 1) in Relion 3.0 from K3 data. The resolution is estimated at FSC=0.143. **(C)** Masks used for multi-body refinement. **(D)** Contributions of eigenvectors to the motion in the Map1. Eigenvectors 1, 2 and 3 contribute to 55% of total motion though only vector 2 shows significant conformational change in Ssl2. **(E)** Histogram of amplitude along second eigenvector. Binned maps shown volume series in Supplementary Movie 4.

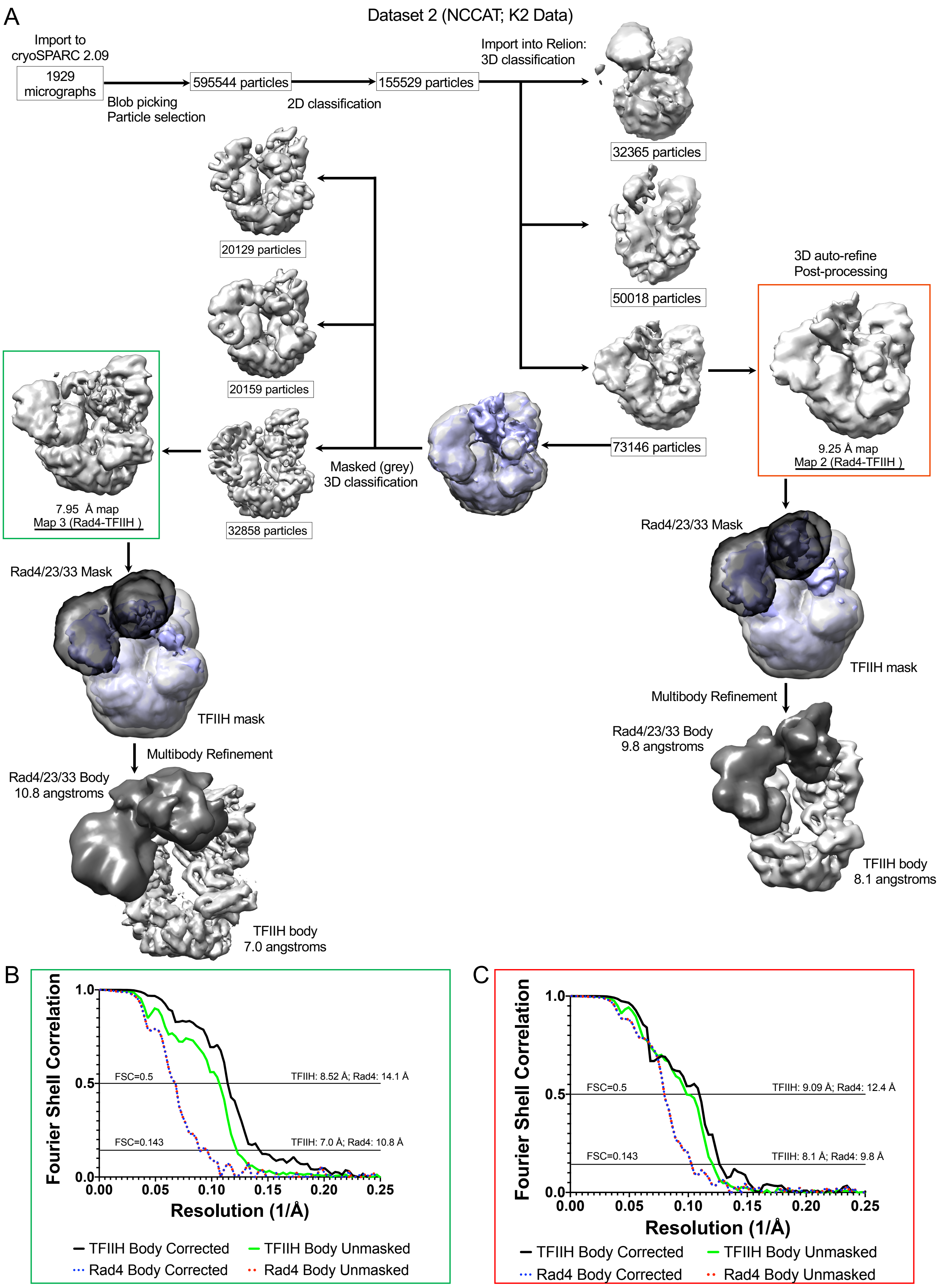
A
Dataset 2 (NCCAT; K2 Data)
Import to
cryoSPARC 2.09
1929
micrographs
Blob picking
Particle selection
595544 particles
2D classification
155529 particles
Import into Relion:
3D classification
32365 particles
50018 particles
73146 particles
3D auto-refine
Post-processing
9.25 Å map
Map 2 (Rad4-TFIIH)
20129 particles
20159 particles
32858 particles
Masked (grey)
3D classification
7.95 Å map
Map 3 (Rad4-TFIIH)
Rad4/23/33 Mask
TFIIH mask
Multibody Refinement
Rad4/23/33 Body
10.8 angstroms
TFIIH body
7.0 angstroms
Rad4/23/33 Mask
TFIIH mask
Multibody Refinement
Rad4/23/33 Body
9.8 angstroms
TFIIH body
8.1 angstroms
B
Fourier Shell Correlation
FSC=0.5
TFIIH: 8.52 Å; Rad4: 14.1 Å
FSC=0.143
TFIIH: 7.0 Å; Rad4: 10.8 Å
Resolution (1/Å)
TFIIH Body Corrected
TFIIH Body Unmasked
Rad4 Body Corrected
Rad4 Body Unmasked
C
Fourier Shell Correlation
FSC=0.5
TFIIH: 9.09 Å; Rad4: 12.4 Å
FSC=0.143
TFIIH: 8.1 Å; Rad4: 9.8 Å
Resolution (1/Å)
TFIIH Body Corrected
TFIIH Body Unmasked
Rad4 Body Corrected
Rad4 Body Unmasked

Supplementary Figure 5.
(A) Summary of data processing procedures and workflow of TFIIH/Rad4-23-33/DNA complex from K2 data collection. Similar to processing of K3 data (A) but focused on TFIIH/Rad4-23-33/DNA complex and conducted in Relion 3.1. **(B-C)** Fourier shell correlation (FSC) curves of Map 2 (B) and Map 3 (C).

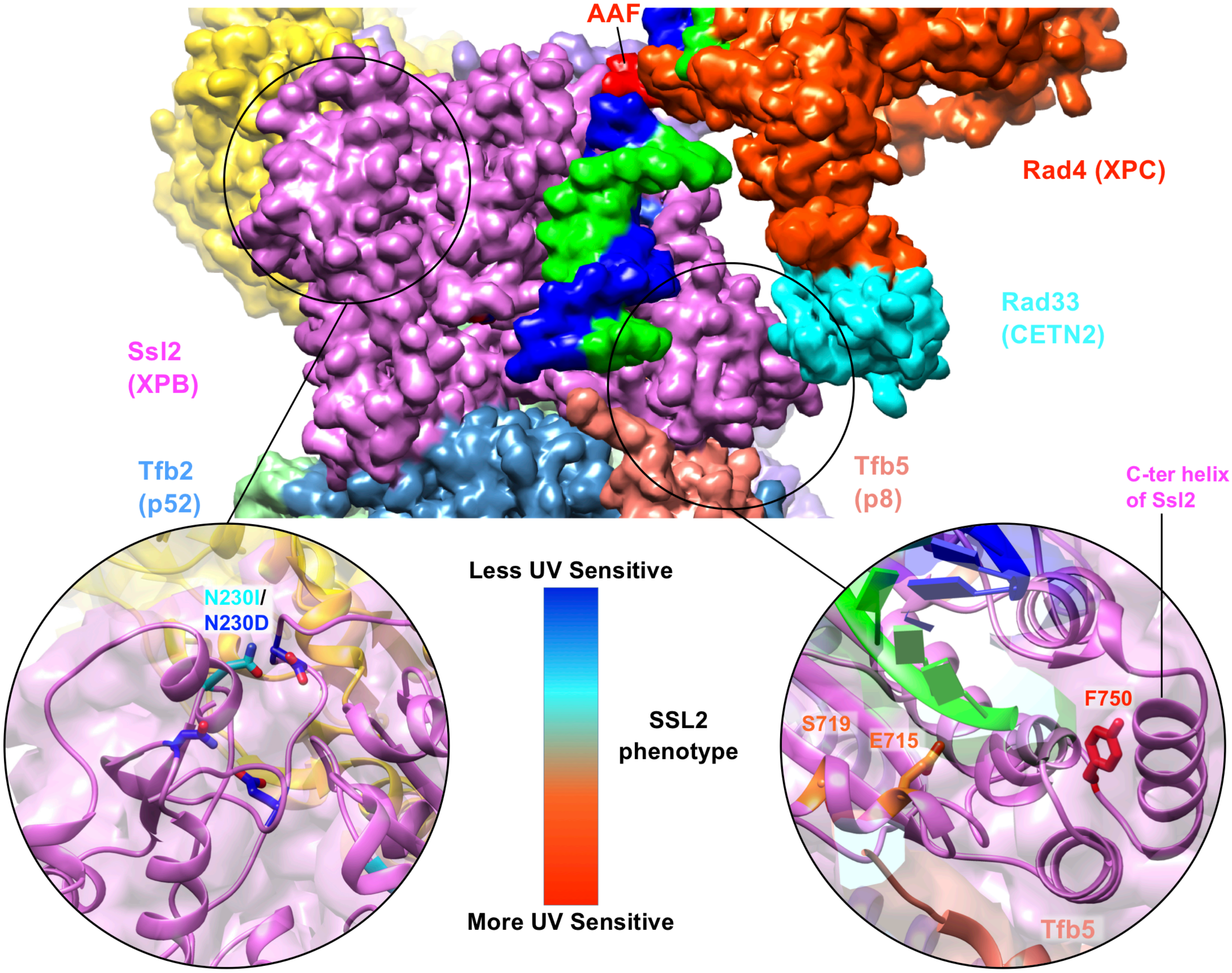

`

Supplementary Figure 6.
Mutations of Ssl2 at the interface with Rad33 and Tfb5 confer UV sensitivity. Mapping of mutations (red, severe; blue, mild) in Fig. 5D onto the structure of the TFIIH/Rad4-23-33/DNA complex.

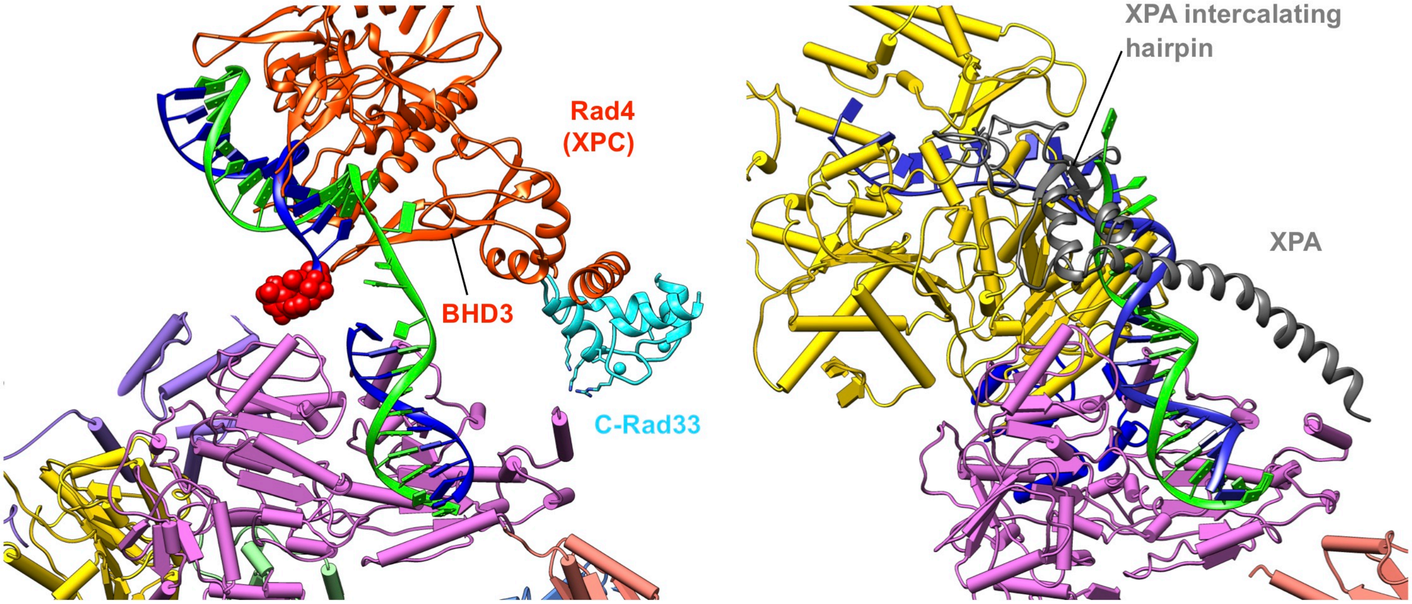

Supplementary Figure 7.
DNA binding sites of the yeast pre-unwound state (left) and previous structure of the human (artificially) unwound state (right, PDB:6RO4).

Supplementary Table 1. Cryo-EM data collection, refinement and validation statistics

	Map 1 (EMDB-22587) (PDB 7K01)	Map 2 (EMDB-22588) (PDB 7K04)	Map 3 (EMDB-22576) (PDB 7M2U)
**Data collection and processing**			
Magnification	105,000x	130,000x	130,000x
Voltage (kV)	300	300	300
Electron exposure (e–/Å^2)	50	42	40
Defocus range (μm)	0.8-2.8	0.8-2.8	0.8-2.8
Pixel size (Å)	0.83	1.08	1.08
Symmetry imposed	N/A	N/A	N/A
Initial particle images (no.)	1,159,858	595,544	595,544
Final particle images (no.)	56,101	73,146	32,858
Map resolution (Å)<br>FSC threshold 0.143 (0.5)	3.86 (4.5)	TFIIH: 8.0 (9.09)<br>Rad4: 9.8 (12.4)	TFIIH: 7.01 (8.53)<br>Rad4: 10.8 (14.1)
Map resolution range (Å)	3.0-12.0		
**Refinement**			
Initial model used (PDB code)	5OQJ/6NMI	5OQJ/6NMI/6CFI	5OQJ/6NMI/6CFI
Map sharpening *B* factor (Å^2)	-77	N/A	N/A
Model composition			
Non-hydrogen atoms	21,058	33,338	23,553
Protein residues	2,795	3,506	2,967
Ligands	6	8	8
R.m.s. deviations			
Bond lengths (Å)	0.008	0.006	0.005
Bond angles (°)	0.825	1.048	0.924
Validation			
MolProbity score	3.39	3.16	3.32
Clashscore	26.94	18.51	23.35
Poor rotamers (%)	11.56	10.06	11.30
Ramachandran plot			
Favored (%)	85.04	86.67	85.22
Allowed (%)	14.81	10.06	14.54
Disallowed (%)	0.15	0.23	0.24

Supplementary Table 2. Oligonucleotides/Primer sets used in this work.

-24/+24 AAF	Fw: 5'-CCCATTCCAACCATCCCACTACCTGTCCATCTTCACTCAACTATCACCC-3'
-24/+24 AAF	Rv: 5'-GGGTGATAGTTGAGTGAAGATGGACAGGTAGTGGGATGGTTGGAATGGG-3'
-14/+14 AAF	Fw: 5'-CCATCCCACTACCTGTCCATCTTCACTCA-3'
-14/+14 AAF	Rv: 5'-TGAGTGAAGATGGACAGGTAGTGGGATGG-3'
-15/+24 AAF	Fw: 5'-CATCCCACTACCTGTCCATCTTCACTCAACTATCACCC-3'
-15/+24 AAF	Rv: 5'-GGGTGATAGTTGAGTGAAGATGGACAGGTAGTGGGATG-3'
-24/+15 AAF	Fw: 5'-CCCATTCCAACCATCCCACTACCTGTCCATCTTCACTCA-3'
-24/+15 AAF	Rv: 5'-TGAGTGAAGATGGACAGGTAGTGGGATGGTTGGAATGGG-3'
-24*/+24 AAF	Fw: 5'-CCCATTCCAACCATCCCACTACCTGTCCATCTTCACTCAACTATCACCC-3'
-24*/+24 AAF	Rv: 5'-GGGTGATAGTTGAGTGAAGATGGACAGGTAGTGGGATGGT-3'
Rad2	Fw: 5'-ATATATAGGATCCATGGGTGTGCATTCATTTTGGGATATTGC-3'
Rad2	Rv: 5'-GCGATCTCGAGTTACATCTTTCTTTTCTTTAGTTTACC-3'
Rad14	Fw: 5'-GCGATATGAATTCGAGGCTAACAGGAAATTAGCAAA
Rad14	Rv: 5'-TATAGAGCGGCCGCTTAAATGTCAATTTCTTC-3'
Rad23	Fw: 5'-GCGTCATGATTAGCTTAACCTTTAAAAATTTCAAGAAGG-3'
Rad23	Rv: 5'-ATACCATGGGTCGGCATGATCGCTGAATAGAATAT-3'
Ssl2TAP	Fw: 5'-CATTAATCAGAAAGATGTATTATAAGAATTTGAAGAAGAATATGTCTTTAATCAATATG-3'
Ssl2TAP	Rv:5'-AATAGATTCAAAATAGGAAGGTGACAATGAAACCAAGCCTATTACGACTCACTATAGGG-3'

Supplementary References

1 Fujiwara, R. & Murakami, K. In vitro reconstitution of yeast RNA polymerase II transcription initiation with high efficiency. *Methods* **159-160,** 82–89 (2019).

2 Zimmet, A. *et al.* Cryo-EM structure of NPF-bound human Arp2/3 complex and activation mechanism. *Sci Adv.* **6,** eaaz7651, doi:10.1126/sciadv.aaz7651 (2020).

3 van Eeuwen, T. *et al.* Structure of TFIIK for phosphorylation of CTD of RNA polymerase II. *Sci Adv.* **7***,* eabd4420, doi:10.1126/sciadv.abd4420 (2021).
