# Supplementary material for: Cryo-EM structure of TFIIH/Rad4–Rad23–Rad33 in damaged DNA opening in nucleotide excision repair
Source: Nat Commun. 2021 Jun 7;12:3338. doi: 10.1038/s41467-021-23684-x (PMC8184850; doi:10.1038/s41467-021-23684-x)
Supplement: Supplementary file 2 — Description of Additional Supplementary Files [file 41467_2021_23684_MOESM2_ESM.pdf]

Description of Additional Supplementary Files

Supplementary Data 1: Full list of identified BS3 crosslinked peptides from the Rad4- 23-33/TFIIH/AAF complex.

Supplementary Movie 1: 3.9A-resolution cryo-EM map (Map 1) of TFIIH and corresponding model. Color scheme corresponds to Figure 3.

Supplementary Movie 2: Comparison between structures of TFIIH in transcription (PDB:5OQJ) and NER (PDB:7K01, this study). For transcription, template and non-template strands are blue and green. For NER, damaged and undamaged strand are blue and green.

Supplementary Movie 3: A comparison between structures of yeast TFIIH/Rad4-23-33/DNA (PDB:7K04) and human TFIIH/XPA/open DNA (PDB:6RO4). Color scheme corresponds to Figure 6.

Supplementary Movie 4: Multibody refinement of Map 1. A ~30 Å movement of Ssl2/Tfb5/Tfb2C brings the damaged strand into close proximity to the ssDNA binding cleft of Rad3.
