# Supplementary material for: Cryo-EM structure of TFIIH/Rad4–Rad23–Rad33 in damaged DNA opening in nucleotide excision repair
Source: Nat Commun. 2021 Jun 7;12:3338. doi: 10.1038/s41467-021-23684-x (PMC8184850; doi:10.1038/s41467-021-23684-x)
Supplement: Supplementary file 3 — Supplementary Data 1 [file 41467_2021_23684_MOESM3_ESM.pdf]

Protein1	Protein2	Residue 1	Residue 2	Relative Score
		**Rad4-Rad23-Rad33 crosslinks**		
Rad23	Rad23	56	49	142
Rad23	Rad23	63	54	117
Rad23	Rad23	75	79	146
Rad23	Rad23	75	54	27
Rad23	Rad23	75	80	171
Rad23	Rad4	13	28	115
Rad23	Rad4	80	404	176
Rad33	Rad4	128	15	142
Rad33	Rad4	128	20	155
Rad33	Rad4	128	28	148
Rad33	Rad4	132	668	183
Rad4	Rad23	24	79	128
Rad4	Rad23	28	79	58
Rad4	Rad23	36	77	45
Rad4	Rad23	36	79	166
Rad4	Rad23	598	79	156
Rad4	Rad23	598	80	87
Rad4	Rad23	616	54	145
Rad4	Rad33	28	128	148
Rad4	Rad4	15	48	158
Rad4	Rad4	15	660	170
Rad4	Rad4	15	28	149
Rad4	Rad4	28	48	106
Rad4	Rad4	28	36	139
Rad4	Rad4	28	45	41
Rad4	Rad4	28	477	105
Rad4	Rad4	28	661	179
Rad4	Rad4	28	20	151
Rad4	Rad4	36	15	95
Rad4	Rad4	36	28	36
Rad4	Rad4	36	477	142
Rad4	Rad4	36	24	149
Rad4	Rad4	44	48	47
Rad4	Rad4	46	44	137
Rad4	Rad4	48	44	182
Rad4	Rad4	48	477	101
Rad4	Rad4	48	28	128
Rad4	Rad4	48	45	224

Rad4	Rad4	48	36	175
Rad4	Rad4	165	369	185
Rad4	Rad4	187	204	183
Rad4	Rad4	204	345	170
Rad4	Rad4	204	346	83
Rad4	Rad4	205	198	161
Rad4	Rad4	212	204	120
Rad4	Rad4	240	247	26
Rad4	Rad4	240	244	182
Rad4	Rad4	240	242	113
Rad4	Rad4	240	245	121
Rad4	Rad4	256	244	162
Rad4	Rad4	256	170	120
Rad4	Rad4	257	170	157
Rad4	Rad4	307	345	156
Rad4	Rad4	326	345	106
Rad4	Rad4	336	346	150
Rad4	Rad4	336	345	181
Rad4	Rad4	336	204	148
Rad4	Rad4	345	477	42
Rad4	Rad4	346	477	148
Rad4	Rad4	346	204	170
Rad4	Rad4	385	350	184
Rad4	Rad4	400	404	126
Rad4	Rad4	400	484	27
Rad4	Rad4	400	492	111
Rad4	Rad4	400	491	182
Rad4	Rad4	442	477	177
Rad4	Rad4	454	492	96
Rad4	Rad4	456	491	47
Rad4	Rad4	456	504	166
Rad4	Rad4	460	492	139
Rad4	Rad4	460	484	185
Rad4	Rad4	460	504	195
Rad4	Rad4	460	507	57
Rad4	Rad4	464	480	177
Rad4	Rad4	468	491	201
Rad4	Rad4	468	504	189
Rad4	Rad4	468	477	91
Rad4	Rad4	468	480	26

Rad4	Rad4	468	484	95
Rad4	Rad4	470	345	162
Rad4	Rad4	470	477	190
Rad4	Rad4	470	480	162
Rad4	Rad4	480	511	168
Rad4	Rad4	480	474	128
Rad4	Rad4	492	484	118
Rad4	Rad4	504	477	164
Rad4	Rad4	521	610	81
Rad4	Rad4	521	514	170
Rad4	Rad4	521	45	115
Rad4	Rad4	521	44	149
Rad4	Rad4	521	492	103
Rad4	Rad4	521	48	159
Rad4	Rad4	598	606	165
Rad4	Rad4	603	36	110
Rad4	Rad4	603	24	83
Rad4	Rad4	660	15	95
Rad4	Rad4	668	15	175
Rad4	Rad4	677	15	169

Protein1	Protein2	Residue 1	Residue 2	Relative Score
**TFIIH-TFIIH crosslinks**				
Rad3	Rad3	2	95	40
Rad3	Rad3	2	94	153
Rad3	Rad3	30	486	173
Rad3	Rad3	30	752	154
Rad3	Rad3	30	476	142
Rad3	Rad3	30	481	147
Rad3	Rad3	30	477	176
Rad3	Rad3	92	95	199
Rad3	Rad3	94	180	144
Rad3	Rad3	112	125	126
Rad3	Rad3	112	77	37
Rad3	Rad3	121	132	90
Rad3	Rad3	121	125	165
Rad3	Rad3	122	132	112
Rad3	Rad3	122	125	181
Rad3	Rad3	125	588	109
Rad3	Rad3	125	110	113

Rad3	Rad3	142	125	157
Rad3	Rad3	276	276	155
Rad3	Rad3	276	142	156
Rad3	Rad3	276	125	133
Rad3	Rad3	336	276	40
Rad3	Rad3	347	344	174
Rad3	Rad3	347	276	22
Rad3	Rad3	447	476	49
Rad3	Rad3	605	112	146
Rad3	Rad3	605	125	205
Rad3	Rad3	605	636	54
Rad3	Rad3	616	588	202
Rad3	Rad3	673	489	81
Rad3	Rad3	673	588	148
Rad3	Rad3	689	636	182
Rad3	Rad3	691	481	128
Rad3	Rad3	691	636	114
Rad3	Rad3	746	728	180
Rad3	Rad3	752	30	106
Rad3	Rad3	752	476	187
Rad3	Rad3	752	2	95
Rad3	Ssl1	30	52	59
Rad3	Ssl1	276	420	87
Rad3	Ssl1	476	52	166
Rad3	Ssl1	737	113	110
Rad3	Ssl1	740	113	118
Rad3	Ssl1	750	113	181
Rad3	Tfb1	94	580	199
Rad3	Tfb4	2	105	149
Rad3	Tfb4	94	102	77
Rad3	Tfb4	276	103	145
Ssl1	Rad3	52	636	136
Ssl1	Rad3	420	276	128
Ssl1	Ssl1	52	420	149
Ssl1	Ssl1	88	420	100
Ssl1	Ssl1	113	52	68
Ssl1	Ssl1	113	420	82
Ssl1	Ssl1	139	201	164
Ssl1	Ssl1	198	201	96
Ssl1	Ssl1	289	113	191

Ssl1	Ssl1	295	315	155
Ssl1	Ssl1	306	315	66
Ssl1	Ssl1	312	113	181
Ssl1	Ssl1	312	118	228
Ssl1	Ssl1	395	414	199
Ssl1	Ssl1	397	420	159
Ssl1	Ssl1	397	414	84
Ssl1	Ssl1	397	425	215
Ssl1	Ssl1	405	420	47
Ssl1	Ssl1	408	420	135
Ssl1	Ssl1	408	414	27
Ssl1	Ssl1	414	420	113
Ssl1	Ssl1	414	397	34
Ssl1	Ssl1	420	52	146
Ssl1	Ssl1	420	420	176
Ssl1	Tfb1	201	255	49
Ssl1	Tfb1	205	255	129
Ssl1	Tfb1	354	189	194
Ssl1	Tfb1	354	512	139
Ssl1	Tfb2	198	415	139
Ssl1	Tfb2	201	418	150
Ssl1	Tfb2	205	418	165
Ssl1	Tfb4	326	323	181
Ssl1	Tfb4	397	103	189
Ssl1	Tfb4	414	99	115
Ssl1	Tfb4	414	97	45
Ssl2	Rad3	228	636	159
Ssl2	Ssl1	222	52	130
Ssl2	Ssl2	64	71	38
Ssl2	Ssl2	65	71	185
Ssl2	Ssl2	88	71	191
Ssl2	Ssl2	88	73	82
Ssl2	Ssl2	90	65	117
Ssl2	Ssl2	90	523	176
Ssl2	Ssl2	371	224	152
Ssl2	Ssl2	372	222	191
Ssl2	Ssl2	791	796	196
Ssl2	Ssl2	791	795	66
Ssl2	Ssl2	822	827	167
Ssl2	Ssl2	824	796	192

Ssl2	Ssl2	827	796	86
Ssl2	Ssl2	827	835	179
Ssl2	Ssl2	835	842	220
Ssl2	Ssl2	835	843	151
Ssl2	Ssl2	835	824	171
Ssl2	Ssl2	843	796	47
Ssl2	Tfb1	335	73	107
Tfb1	Rad3	120	276	158
Tfb1	Rad3	120	125	161
Tfb1	Rad3	291	276	36
Tfb1	Rad3	334	588	168
Tfb1	Rad3	376	125	188
Tfb1	Rad3	390	588	94
Tfb1	Rad3	393	588	43
Tfb1	Rad3	394	588	108
Tfb1	Rad3	396	588	158
Tfb1	Rad3	401	588	156
Tfb1	Rad3	405	588	24
Tfb1	Rad3	428	95	98
Tfb1	Ssl1	120	397	176
Tfb1	Ssl1	120	201	55
Tfb1	Ssl1	171	315	154
Tfb1	Ssl1	189	201	114
Tfb1	Ssl1	420	278	191
Tfb1	Ssl1	427	113	80
Tfb1	Ssl1	444	113	122
Tfb1	Ssl1	451	420	29
Tfb1	Ssl2	186	796	157
Tfb1	Tfb1	55	101	137
Tfb1	Tfb1	73	279	195
Tfb1	Tfb1	73	183	143
Tfb1	Tfb1	73	267	92
Tfb1	Tfb1	73	71	193
Tfb1	Tfb1	73	69	154
Tfb1	Tfb1	73	189	188
Tfb1	Tfb1	73	171	168
Tfb1	Tfb1	73	268	176
Tfb1	Tfb1	83	279	59
Tfb1	Tfb1	83	71	172
Tfb1	Tfb1	83	69	69

Tfb1	Tfb1	83	70	162
Tfb1	Tfb1	109	268	168
Tfb1	Tfb1	111	279	121
Tfb1	Tfb1	112	69	202
Tfb1	Tfb1	117	171	123
Tfb1	Tfb1	120	173	191
Tfb1	Tfb1	120	279	102
Tfb1	Tfb1	120	71	119
Tfb1	Tfb1	120	70	168
Tfb1	Tfb1	120	580	162
Tfb1	Tfb1	120	65	128
Tfb1	Tfb1	120	268	80
Tfb1	Tfb1	120	69	185
Tfb1	Tfb1	120	171	110
Tfb1	Tfb1	120	186	90
Tfb1	Tfb1	120	458	157
Tfb1	Tfb1	171	581	69
Tfb1	Tfb1	183	70	31
Tfb1	Tfb1	183	120	160
Tfb1	Tfb1	186	71	174
Tfb1	Tfb1	186	70	154
Tfb1	Tfb1	189	120	93
Tfb1	Tfb1	189	70	195
Tfb1	Tfb1	225	267	93
Tfb1	Tfb1	227	267	46
Tfb1	Tfb1	252	255	193
Tfb1	Tfb1	267	71	190
Tfb1	Tfb1	267	120	219
Tfb1	Tfb1	267	70	149
Tfb1	Tfb1	276	71	153
Tfb1	Tfb1	276	120	90
Tfb1	Tfb1	276	267	86
Tfb1	Tfb1	279	71	133
Tfb1	Tfb1	279	70	167
Tfb1	Tfb1	291	295	168
Tfb1	Tfb1	300	290	47
Tfb1	Tfb1	300	291	34
Tfb1	Tfb1	324	268	158
Tfb1	Tfb1	334	267	190
Tfb1	Tfb1	334	393	44

Tfb1	Tfb1	334	394	173
Tfb1	Tfb1	396	390	155
Tfb1	Tfb1	401	173	46
Tfb1	Tfb1	401	390	199
Tfb1	Tfb1	402	173	62
Tfb1	Tfb1	438	444	91
Tfb1	Tfb1	438	428	214
Tfb1	Tfb1	444	458	24
Tfb1	Tfb1	444	438	153
Tfb1	Tfb1	445	458	142
Tfb1	Tfb1	446	438	31
Tfb1	Tfb1	458	438	203
Tfb1	Tfb1	574	171	200
Tfb1	Tfb1	578	171	84
Tfb1	Tfb1	580	268	163
Tfb1	Tfb4	71	323	133
Tfb1	Tfb4	73	323	141
Tfb1	Tfb4	171	323	149
Tfb1	Tfb4	444	277	73
Tfb1	Tfb4	451	323	142
Tfb1	Tfb4	458	144	103
Tfb1	Tfb4	515	207	28
Tfb1	Tfb4	578	323	174
Tfb1	Tfb4	580	332	132
Tfb2	Rad3	237	276	186
Tfb2	Ssl1	313	420	102
Tfb2	Ssl1	313	201	80
Tfb2	Ssl1	326	113	190
Tfb2	Ssl1	326	414	128
Tfb2	Ssl1	326	417	159
Tfb2	Ssl1	415	201	109
Tfb2	Ssl1	418	201	158
Tfb2	Ssl1	419	201	163
Tfb2	Ssl2	168	71	198
Tfb2	Ssl2	170	71	174
Tfb2	Tfb2	80	506	173
Tfb2	Tfb2	262	238	146
Tfb2	Tfb2	326	284	110
Tfb2	Tfb2	419	415	159
Tfb2	Tfb2	487	489	182

Tfb2	Tfb2	488	490	164
Tfb2	Tfb2	490	488	142
Tfb2	Tfb2	499	171	81
Tfb2	Tfb2	506	511	62
Tfb2	Tfb4	238	144	141
Tfb2	Tfb4	238	103	169
Tfb2	Tfb4	262	103	131
Tfb2	Tfb4	267	102	34
Tfb2	Tfb4	283	174	85
Tfb2	Tfb4	284	173	133
Tfb2	Tfb4	313	174	44
Tfb2	Tfb4	326	173	32
Tfb2	Tfb4	326	207	118
Tfb2	Tfb4	326	103	211
Tfb2	Tfb4	343	173	163
Tfb2	Tfb4	419	174	135
Tfb2	Tfb4	506	167	54
Tfb2	Tfb5	495	51	188
Tfb2	Tfb5	499	6	201
Tfb2	Tfb5	506	6	149
Tfb4	Rad3	102	276	137
Tfb4	Rad3	102	125	182
Tfb4	Ssl1	95	414	192
Tfb4	Ssl1	95	397	195
Tfb4	Ssl1	99	414	123
Tfb4	Ssl1	99	397	174
Tfb4	Ssl1	102	420	149
Tfb4	Ssl1	102	113	176
Tfb4	Ssl1	103	397	43
Tfb4	Ssl1	138	397	193
Tfb4	Ssl1	319	315	69
Tfb4	Ssl1	319	326	138
Tfb4	Ssl1	321	315	130
Tfb4	Tfb1	319	171	62
Tfb4	Tfb1	321	171	113
Tfb4	Tfb2	105	238	189
Tfb4	Tfb2	108	238	99
Tfb4	Tfb4	84	138	179
Tfb4	Tfb4	87	102	168
Tfb4	Tfb4	95	144	140

Tfb4	Tfb4	95	138	44
Tfb4	Tfb4	99	138	178
Tfb4	Tfb4	102	137	30
Tfb4	Tfb4	105	102	182
Tfb4	Tfb4	108	103	42
Tfb4	Tfb4	115	102	183
Tfb4	Tfb4	137	102	79
Tfb4	Tfb4	137	103	166
Tfb4	Tfb4	138	103	89
Tfb4	Tfb4	144	102	96
Tfb4	Tfb4	171	207	155
Tfb4	Tfb4	173	207	56
Tfb4	Tfb4	174	207	56
Tfb4	Tfb4	207	207	116
Tfb4	Tfb4	319	323	156
Tfb4	Tfb4	323	330	62
Tfb4	Tfb4	323	332	59
Tfb5	Ssl2	59	65	123
Tfb5	Tfb2	6	80	90
Tfb5	Tfb4	6	171	155

Protein1	Protein2	Residue 1	Residue 2	Relative Score
**TFIIH/Rad4-Rad23-Rad33 crosslinks**				
Rad23	Tfb4	54	102	97
Rad33	Ssl2	58	796	175
Rad4	Ssl1	379	414	139
Ssl2	Rad4	624	36	127
Tfb1	Rad23	120	54	223
Tfb1	Rad4	73	48	147
Tfb1	Rad4	109	24	167
Tfb1	Rad4	120	48	102
Tfb1	Rad4	120	36	41
Tfb1	Rad4	120	477	163
Tfb1	Rad4	243	48	49
Tfb1	Rad4	279	477	134
Tfb1	Rad4	279	48	98
Tfb4	Rad4	95	379	122
Tfb4	Rad4	138	379	92
Tfb4	Rad4	323	477	31
